# Addressing the needs of nano-rare patients: the n-Lorem experience

**DOI:** 10.1093/nar/gkag504

**Published:** 2026-06-02

**Authors:** Stanley T Crooke, Sarah Glass, Joseph G Gleeson, Laurence Mignon, Konstantina Skourti-Stathaki, Julie Douville, Megan Knutsen, He Pu, Jennifer M Bain, Elizabeth Berry-Kravis, Neil A Shneider, Olivia Kim-McManus, Florian S Eichler, Wendy K Chung, Amanda Nagy, Horacio Kaufmann, Alejandra Gonzalez-Duarte, Björn Oskarsson, Emily A McCourt, Nelson Leung

**Affiliations:** n-Lorem Foundation, Carlsbad, CA 92101, United States; n-Lorem Foundation, Carlsbad, CA 92101, United States; Department of Neurosciences, University of California, San Diego, CA 92093, United States; Rady Children’s Institute for Genomic Medicine, San Diego, CA 92123. United States; n-Lorem Foundation, Carlsbad, CA 92101, United States; n-Lorem Foundation, Carlsbad, CA 92101, United States; n-Lorem Foundation, Carlsbad, CA 92101, United States; n-Lorem Foundation, Carlsbad, CA 92101, United States; n-Lorem Foundation, Carlsbad, CA 92101, United States; Department of Neurology, Division of Child Neurology, Columbia University Irving Medical Center, New York, NY 10032, United States; Department of Pediatrics, Neurological Sciences and Anatomy and Cell Biology, Rush University Medical Center, Chicago, IL 60612, United States; Center for Motor Neuron Biology and Disease, Columbia University Irving Medical Center, New York, NY 10032, United States; Department of Neurology, Eleanor and Lou Gehrig ALS Center, Columbia University Irving Medical Center, New York, NY 10032, United States; UCSD Rady Children’s Institute for Genomic Medicine, San Diego, CA 92123, United States; Department of Neurology, Harvard Medical School, Boston, MA 02115, United States; Department of Neurology, Massachusetts General Research Institute, Boston, MA 02114, United States; Department Pediatrics at Boston Children’s Hospital, Boston, MA 02115, United States; Harvard Medical School, Boston, MA 02115, United States; Department of Neurology, Harvard Medical School, Boston, MA 02115, United States; Department of Neurology, Massachusetts General Research Institute, Boston, MA 02114, United States; Department of Neurology, New York University School of Medicine, New York, NY 10017, United States; Department of Neurology, Dysautonomia Center, NYU Langone Health, NYU Grossman school of Medicine, New York, NY 10016, United States; Mayo Clinic, Jacksonville, FL 55905, United States; Department of Ophthalmology, University of Colorado School of Medicine, Children’s Hospital of Colorado, Anschutz Medical Campus, Aurora, CO 80045, United States; Division of Nephrology and Hypertension, Department of Internal Medicine, Mayo Clinic, Rochester, MN 55905, United States; Division of Hematology, Department of Internal Medicine, Mayo Clinic, Rochester, MN 55905, United States

## Abstract

Patients with extremely rare pathogenic variants pose unique challenges to current healthcare systems. Nano-rare mutations have been defined as mutations with a known prevalence of <30 patients worldwide, but because of the small fraction of humans who have undergone genetic testing, neither the precise prevalence of individual mutations nor the total prevalence of patients with nano-rare mutations is known. n-Lorem is a non-profit founded in 2020 with the mission of equitably discovering, developing, and providing bespoke experimental antisense oligonucleotides (ASOs) for free, for life, to patients with nano-rare mutations that are amenable to ASO treatment. In this perspective, we provide an overview of the n-Lorem processes and systems, the characteristics of the first 329 patients who have applied for treatment for whom initial assessment was completed and suitability for ASO treatment determined, and a summary of the results of ASO treatment for patients treated to date. Detailed data on individual patients and the overall clinical safety and tolerability profiles of the ASOs for which there are clinical data are the subjects of other manuscripts.

## Graphical Abstract

**Graphical Abstract:**
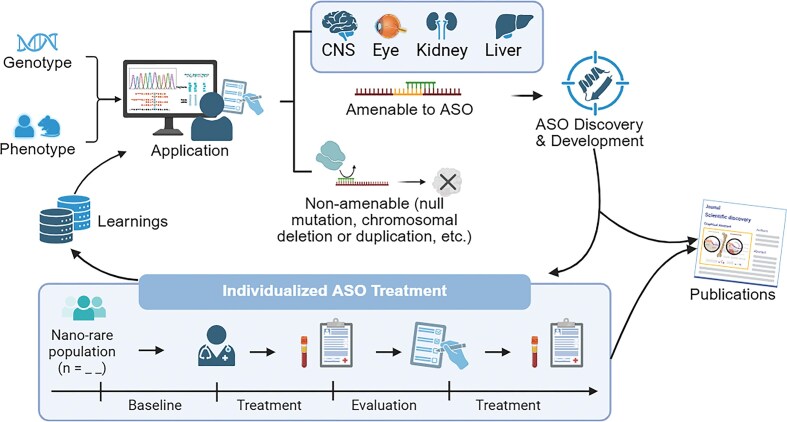

## Introduction

Nano-rare mutations have been defined as pathogenic mutations with a known world-wide prevalence of <30 [1, 2]. However, because of the small fraction of humans who have undergone genetic testing, neither the precise prevalence of individual mutations nor the overall prevalence of these extremely rare pathogenic mutations is known. n-Lorem is a non-profit founded in 2020 with the mission of equitably providing experimental antisense oligonucleotides (ASOs) for free, for life, to patients with nano-rare mutations [3, 4]. To achieve this mission, we have taken advantage of the rapidity, efficiency, and versatility of ASO technology and the unique guidance issued by the FDA that supports treating nano-rare patients with experimental ASOs of well-understood chemical classes with only *in vitro* pharmacological data and a single 3-month toxicology study conducted in compliance with Good Laboratory Practice (GLP)[5–18].

Our goals at n-Lorem are to respond to the urgent needs of nano-rare patients by providing experimental ASOs for those patients amenable to ASO treatment while creating scalable systems and processes that assure that only appropriate patients are treated, maximize the safety and tolerability of ASO treatments, and what we learn from each patient and our aggregate experience. We are also collaborating with other organizations attempting to establish a more holistic solution to this emergent healthcare issue. Our specific objectives are to mount a new ASO discovery and development program for each patient, initiate treatment within 18–24 months after acceptance, and provide lifetime treatment to each patient for free for life. The cost to n-Lorem to treat a patient for life is approximately $1 million. Lastly, we are attempting to create a community that provides support and education by establishing a podcast series dedicated to the needs of nano-rare patients and their families, providing “Lessons in Antisense” and hosting annual meetings for the scientific and medical communities, as well as nano-rare patients and families [19].

In this perspective, we summarize the n-Lorem process and our industrialized systems, our overall experience, a number of important lessons learned, and challenges that remain. A detailed presentation of the safety and tolerability observed in n-Lorem-sponsored clinical trials has been submitted for publication [5]. As this perspective is designed to provide an overview of our experience and we are providing detailed data on individual patients and groups of patients in other publications [20, 21], here we provide a summary of our clinical experience.

### FDA guidance

The FDA guidance for IND (Investigational New Drug) submissions for individualized ASOs [14–17, 18] is predicated on >30 years of experience with phosphorothioate (PS) ASOs of various chemical classes developed at Ionis (and now n-Lorem), including the publication of safety databases for PS 2′-methoxyethyl (MOE) and PS 2′-MOE ASOs with *N*-acetyl-galactosamine (GalNAc) conjugates [10–13]. Though the guidance does not specify what chemical classes have been adequately studied, we typically use PS 2′-MOE “gapmer” ASOs designed to serve as RNase H1 substrates once bound to a target RNA. We also use fully 2′-modified PS MOE ASOs to alter RNA intermediary metabolism (5′ capping, polyadenylation, splicing, translation, transportation, modification, or metabolism). Meaningful clinical experience with PS 2′-constrained ethyl ASOs has also been reported, but n-Lorem has not yet submitted an IND application for an ASO of that chemical class [10–12].

One of the several major advantages of ASO technology is that within a chemical class, the basic pharmacokinetic/pharmacodynamic (PK/PD) properties are similar irrespective of the sequence of the PS ASO. Distribution of PS ASOs is driven by PS content and is consistent so long as a minimum of 10 PS are present. Tissue elimination half-life is very modestly affected by sequence, but not sufficiently to require adjustments of dose schedule. As long as an optimal ASO has been selected, the range of doses is narrow enough to use a standard starting dose, dose escalation scheme, and maximum dose. Thus, based on past experience in many thousands of patients, we can select a route of administration, an appropriate range of doses, and administration schedules. Furthermore, synthetic methods and analytical processes are well established and apply to all PS ASOs that are of a particular chemical class, facilitating rapid and relatively inexpensive advancement of candidate PS ASOs to the clinic. Lastly, if stored properly, PS ASOs are quite stable and this supports manufacturing sufficient quantities of each PS ASO to treat patients for many years.

The guidance supports rapidly treating very sick nano-rare patients with only *in vitro* pharmacological data and a single 3-month rodent GLP toxicology study. As of July 31, 2025, under this guidance, n-Lorem has had >30 INDs approved by the two neurological divisions, the ophthalmological division and the cardio-renal division of the FDA, as well as one clinical trial application (IND equivalent) approved by Health Canada. Though there are suggestions that other countries may establish guidance that facilitates ASO treatment and the regulatory authorities in the UK have announced that they are working on guidance for nano-rare patients, to date only the US FDA has issued such guidance.

### The n-Lorem process

**Figure 1.**
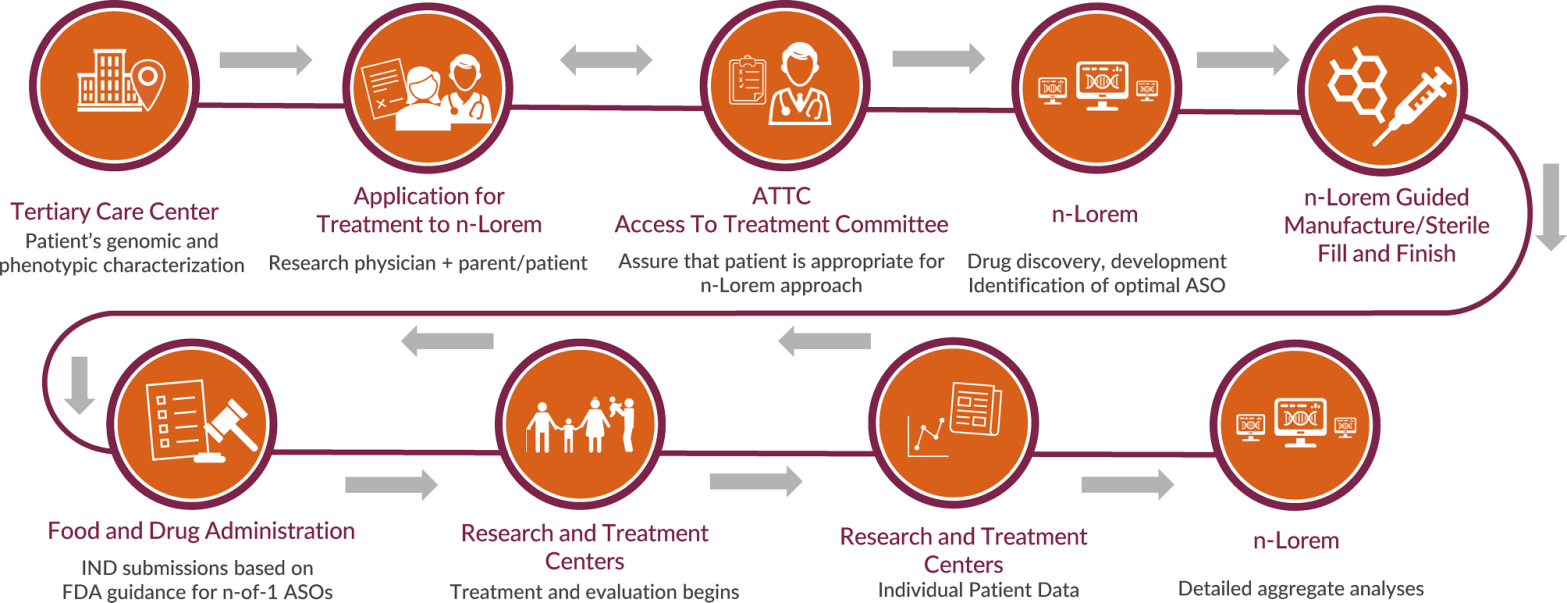
The n-Lorem process from application to IND submission to data collection and analyses.

**Figure 2.**
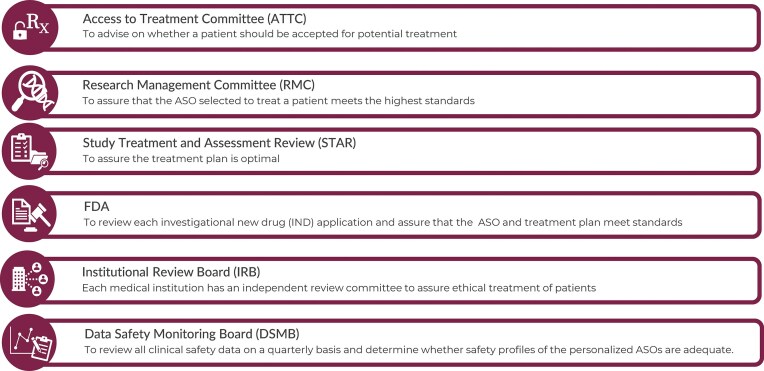
The n-Lorem quality control committees (composition of committees presented in the Supplementary materials).

n-Lorem responds to the needs of individual nano-rare patients and our process is initiated when a research physician submits an application for treatment, which is available on our website (Fig. 1). The n-Lorem process is derived from >35 years of broad experience with PS ASOs of various chemistries, designs, and mechanisms of action in a vast array of cell types, including growing experience in iPSC (induced pluripotent stem cell)- derived human cells, many animal species, a large number of animal models, and hundreds of clinical trials with ASOs administered via intravenous (IV), subcutaneous (SC), intrathecal (IT), intravitreal (IVT), aerosol, intradermally, and topically intended to treat a broad range of diseases (for review, see references 5–13). “In process” quality control is managed by several committees comprised of extramural and n-Lorem experts (Fig. 2). The specific functions and compositions of each committee are provided in the Supplementary materials.

In brief, several critical decisions must be made with regard to every patient application. First, we must determine whether it is appropriate to treat a patient with an ASO. Second, we must determine whether we have an ASO that is satisfactory to administer to a sick patient. Third, we must assure that our treatment goals are focused on significant needs of each patient and that we have prespecified clinical measures to assess whether the patient is benefiting from treatment. Fourth, the safety and tolerability profile for each patient must be assessed quarterly and it must be determined that the risk/benefit profile in each patient justifies continuing treatment quarterly. Lastly, the performance of the entire portfolio is assessed quarterly.

### Determining whether the patient applying for treatment is appropriate to treat with an ASO

**Figure 3.**
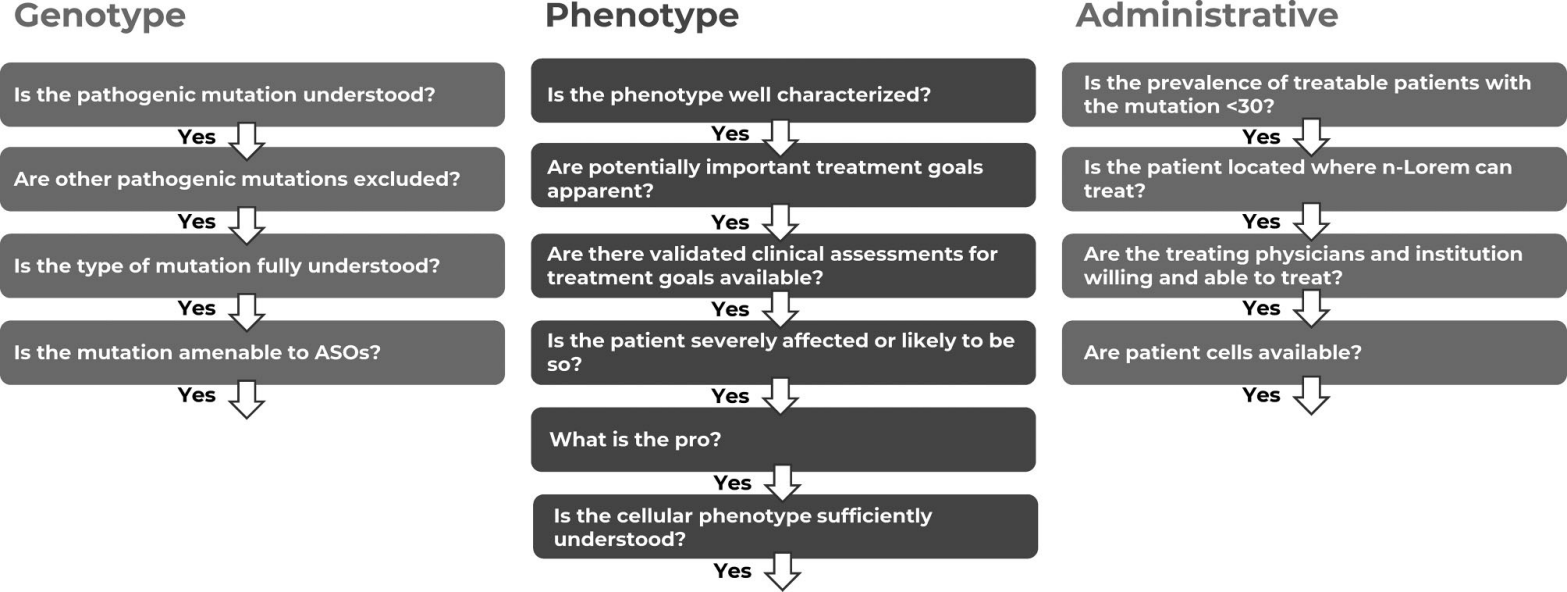
Initial evaluation of applications for treatment. n-Lorem considers a variety of genotypic, phenotypic, and administrative issues.

Upon receipt of an application, each is blinded with regard to patient identity. We then evaluate the genotype and phenotype of the patient to determine whether the mutation is potentially amenable to treatment with ASOs, if the primary manifestations of the mutation are in an organ with which we have the required experience to be comfortable treating with a PS ASO, and finally, determine whether a number of administrative considerations are met satisfactorily (Fig. 3). Next, we work with the submitting physician to define primary, secondary, and exploratory treatment goals and the clinical measures that will be used to assess whether the ASO results in improvement in clinical characteristics that are important to the patient. To make this determination we must thoroughly understand the genotype and phenotype of the patient, the organs affected, whether the patient has access to an appropriate research physician and institution, and meets a number of administrative requirements, as shown in Fig. 3. This requires significant scholarship and consultation with the submitting physician and relevant experts. These steps are performed by senior n-Lorem team members.

Often, additional work is required to prove that the nature of the mutation is well understood. A frequent issue requiring additional work is to prove that the mutation is, in fact, the sole cause of the entire cellular phenotype. Since more than half of our patients require allele-selective RNase H1 ASOs, another question that frequently needs to be addressed is the level of allele-selectivity required. These questions and others can be addressed through iPSC derived from patients, but answering these and other questions can be a cause for delay in making a decision as to whether the patient should be treated with a PS ASO. Rarely, a specific phenotypic characteristic of a loss of function (LOF) mutation may result in delay or rejection of an application for an allele-selective RNase H1 PS ASO targeted to treat a toxic gain of function (TGOF) mutation. An excellent example of this type of issue is a patient with a TGOF mutation in ATPase Na^+^/K^+^ transporting subunit alpha 3 (*ATP1A3*). This gene encodes the catalytic subunit of a sodium channel. It is expressed primarily in the brain and mutations in *ATP1A3* result in clinical syndromes encompassed by the designation Alternating Hemiplegia of Childhood (AHC), a disorder characterized by transient hemiplegia and many other manifestations. Unfortunately, it has also been reported that heterozygous LOF mutations can be associated with abrupt onset, severe, and progressive dystonia [22]. Since it was unclear what level of reduction of wild type (WT) *ATP1A3* might be associated with this phenomenon, after discussing the issue with AHC experts, we had to decline attempting to treat this patient. This example emphasizes the need to integrate a thorough understanding of genotype and phenotype with appropriate concern about patient safety. Advice from experts in the syndrome is also critical to assure that these complex risk/benefit decisions are of the highest quality.

### Presentation of the patient application to ATTC and final decision on acceptance or rejection of application

A formal presentation is then prepared by the n-Lorem team and presented to the ATTC (access to treatment committee), which is charged with providing advice concerning the advisability of treating the patient with an ASO. We try to complete the initial assessment and present the patient to ATTC within 6 weeks of receipt of each application. The ATTC provides advice about complex risk/benefit judgements that are enhanced from the perspectives of many types of experts. The final decision to accept an application is then made by the n-Lorem executive team.

### PS ASO design, discovery, and preclinical development

As ASOs can be designed to take advantage of several post-RNA binding mechanisms, the type of mutation must be clearly defined. This is typically analyzed in iPSC-derived patient cells by assessing the impact of reducing or altering the target on the cellular phenotype, as well as a thorough review of the literature.

Though ASO technology supports rational design and we have mechanistic insights into several of the potential toxicities, PK/PD properties (see references 6 and 7 for review), a multistep screening process that involves significant numbers of PS ASOs at each step is required to assure that an optimal ASO is selected. The n-Lorem design algorithm and screening processes are informed by more than three decades of experience in creating and advancing the technology. The design algorithm prevents the use of problematic di-nucleotides, other motifs, and ASOs designed to bind to repetitive sequences such as poly-pyrimidine tracts, poly-adenine (A) tracts, and Alu sequences. It also excludes sequences that are likely to form internal structures or inter-oligonucleotide structures. Finally, it also excludes guanosine (G)-rich sequences that could form G-quartets [23]. As a review of the literature would suggest, typically PS 2′-MOE ASOs are 18–20 nucleotides in length and gapmer designs usually include a 10-nucleotide PS deoxynucleotide gap flanked by 4 or 5 2′-modified nucleotides at 5′and 3′ flanks of the gap.

Since the complexity of the CNS demands that the screening process for IT-administered PS ASOs for CNS diseases be more elaborate than that for non-CNS diseases and the majority of our patients have neurological disorders, as an example, we will focus on the PS ASO discovery process n-Lorem employs for PS ASOs to be administered IT. Based on >35 years of experience creating, advancing, and validating antisense technology, we consider every step essential. Based on experiments in which the number of ASOs evaluated at each step were systematically varied (for example, in the initial *in vitro* screen to identify active PS 2′-MOE gapmer ASOs, the number of ASOs evaluated were varied from about 100 to many thousands of ASOs necessary to tile the entire *APOB* and the entire *APOC3* pre-mRNA transcripts), we consider the numbers of PS ASOs to be tested at each step to be the minimum number required to consistently identify optimal ASOs suitable for clinical use. Similarly, the analytes measured at each step were selected based on multiple experiments assessing and validating the analytes and all are important to be assessed (Table 1). Patient-derived fibroblasts, iPSCs, or iPSC-derived neurons are used to both identify optimal ASOs and assess the effects of ASO treatment on cellular phenotypes. For RNase H1-activating PS ASOs, a minimum of PS gapmer ASOs designed to bind to 400–500 sites in a target RNA are typically screened at a single dose level. In most cases, the PS ASOs are evaluated in iPSC-derived patient neuronal cells. As a general rule, the single dose level screening identifies >50 potentially attractive PS ASOs, but at a minimum, we screen sites until we identify 50–75 attractive PS ASOs to enter five-point dose response evaluations in the same cells. Such an effort typically identifies 20 or more PS ASOs that are attractive enough to proceed to *in vivo* rat tolerability studies. Prior to initiating *in vivo* testing, an *in silico* analysis of potential hybridization-based off-targets is performed and if off-targets that may result in non-selective effects are identified, potential effects on those targets by the lead PS ASOs are evaluated in the same cells. Attractive PS ASOs are then administered to BJAB cells. BJAB cells are a human B cell line derived from a Burkett Lymphoma patient [24, 25] and the analyte measured is an increase in CCL2 (C-C motif chemokine ligand 2) RNA detected by polymerase chain reaction. The purpose of this step is to identify any PS ASO that may have greater potential to activate the innate immune system (see Supplementary Fig. S1).

**Table 1.** The n-Lorem process to identify optimal PS ASOs for CNS disease

Screening step	Purpose	Approximate minimum numbers of ASOs typically evaluated	Minimum criteria
ASO design including *in silico* off-target assessment	Exclude motifs associated with ASO structure, repeat sequences, cytotoxicity, pro-inflammatory effects and off targetsInclude attractive motifs	Scan entire pre-mRNAApply algorithms	Motifs known to enhance potency include; motifs associated with potential toxicities or non-specific hybridization excluded
Primary ASO screen	To identify optimal sites in target RNA for ASO and H-1 binding	∼500	>80% target reduction
Five-point dose response evaluation of multiple ASOs	To select at least 20 ASOs for *in vivo* tolerability screening	∼50–75	IC50 1 μmol (free uptake)
*In vitro* off-target analysis	To confirm selectivity of ASO for target RNA versus any off-targets that are expressed in relevant cells and have important functions	As many as necessary	∼10-fold difference in IC50s for target RNA versus off target
BJAB Assay	To exclude activators of innate immunity	∼50–75	Less than two-fold increase in TNF-alpha at high ASO concentrations
Single dose tolerability screen in rodents at high dose including histopathology of CNS	To identify optimally tolerated lead ASOs	20	Exclude poorly tolerated candidates and include ASO with an optimal therapeutic index. AIF1 GFAP. Microglia Astrocytes
Repeat dose GLP 3-month rodent toxicity	To identify NOAEL and target organ effects	1–3	A therapeutic index >10 with an acceptable NOAEL
GMP manufacturing	Ensure quality ASO drug substance	1	Pure, stable substance
Sterile fill and finish	Ensure quality, stable, and sterile ASO drug product	1	Sterile vials for administration

AIF1: allograft inflammatory factor 1, GFAP: glial fibrillatory acidic protein

For rodent tolerability and GLP toxicology studies of PS ASOs administered IT, we use the rat because that species can be dosed via that route while the mouse must be dosed by intracerebroventricular delivery. Rat tolerability studies are designed to identify any PS ASOs that may have the potential to cause adverse events (AEs) in humans that were not identified by *in vitro* assays. To assess tolerability, single doses of 3 mg are administered IT and the effects of the PS ASOs are evaluated for 8 weeks post-dose. In the rat after IT dosing, mechanistically there are at least four types of AEs that can occur. Two acute or sub-acute events appear to be related to the small volume of cerebrospinal fluid (CSF) (~300 μl) [20] and the relatively high doses used in these studies. An acute (within hours) sedation-type response is often observed and is thought to be related to high local CSF PS ASO concentrations inhibiting synaptic traffic. As this effect can be ameliorated by reducing PS content, it is likely that disruption of synaptic traffic is secondary to PS ASO binding to synaptic signaling proteins located in the plasma membrane of cells at the synapse [20, 21]. A sub-acute (hours to days) excitatory response that may include seizures is also frequently observed. This is thought to be secondary to chelation of divalent cations (PS ASOs are effective chelators) and the AE can be ameliorated by formulating PS ASOs with magnesium [21]. To date, we have not observed a human clinical response similar to those observations in the rat. However, out of an abundance of caution, we continue to terminate the development of PS ASOs that result in exaggerated acute or sub-acute responses in the rat.

Two other mechanisms are known to cause AEs in rats after IT dosing. Some PS ASOs of all chemical classes can result in direct cytotoxicity. The mechanism accounting for direct cytotoxicity has been shown to be due to forming toxic aggregates containing PS ASOs, RNase H1, and paraspeckle proteins in cells and in animals administered toxic PS ASOs systemically [26, 27]. In the CNS, the precise mechanism is not as clearly established, but Purkinje cells are uniquely sensitive to cytotoxic events in the CNS and Purkinje cell death is a strong indicator of direct cytotoxicity [28]. The most frequently encountered AE in rats and in humans appears to be immune activation, particularly innate immune activation. To assess the risk of immunotoxicity, we evaluate markers of glial cell and astrocyte activation [28, 29].

### Design and discovery of optimal allele-selective PS ASOs

TGOF mutations in essential genes require allele-selective PS ASOs that can serve as substrates for RNase H1 when the ASO is bound to target RNA, i.e. selectively reduce mutant (MT) RNA (and protein) while sparing the WT RNA (and protein). Obviously, this complicates the discovery process and introduces additional requirements that must be addressed if the ASO discovery process is to be successful. Specifically, it is essential to define the minimum allele-selectivity required for the target which can vary depending on a variety of factors. As a general rule, we seek at least five-fold selectivity for MT versus the WT RNA. For some targets, such as ion channels, at least 10-fold selectivity is required. For the majority of targets submitted, the required level of allele-selectivity must be defined by reduction of both the WT RNA in control cells or both the WT and MT RNA reduction in the patient-derived cells, coupled to evaluation of the cellular phenotype. A good example of this is a TGOF mutation in mitogen-activated protein kinase 8 interacting protein 3 (MAPK8IP3), a gene that encodes c-Jun N-terminal kinase (JNK)-interacting protein 3 (JIP3), a multifunctional protein about which relatively little was known. This required quite extensive effort to fully characterize the cellular phenotype and evaluate the effects of MT and WT versus MT-only target reduction in control WT and patient-derived cells WT and MT [30].

To design allele-selective RNase H1 PS ASOs, we usually design PS ASOs to non-pathogenic single nucleotide variants (SNVs) [31]. Therefore, we require long read sequencing and phasing to identify all the SNVs and define whether they are on the MT or WT allele. The more non-pathogenic SNVs, the more likely the success and if two or fewer non-pathogenic SNVs are present in a gene, success in creating an allele-selective ASO is unlikely [19]. The preclinical development steps for an allele-selective ASO are equivalent to a non-allele-selective ASO.

### Discovery and development of optimal PS ASOs to alter RNA splicing

If a patient requires an ASO designed to alter RNA splicing, the entire pre-mRNA transcript is evaluated to identify canonical and non-canonical splice sites. Then, fully 2′-MOE-modified PS ASOs are designed to tile each relevant splice site. The effectiveness of ASOs designed to alter splicing is determined by evaluating RNA processing and the production of the desired protein. Once a PS 2′-MOE ASO that effectively corrects the causative miss-splicing event is identified, preclinical development is similar to that of gapmer PS ASOs designed to reduce target proteins.

### Review and approval of the PS ASO to advance to treat a patient

Once a decision to accept a patient for potential PS ASO treatment is made, the next critical decision is to determine whether the best PS ASO discovered has a profile sufficient to advance it to treat the patient. Important considerations include potency and selectivity for the target gene. For allele-selective RNase H1 PS ASOs, the selectivity for the mutant RNA versus the wild type RNA must be determined. For ASOs designed to alter splicing, we assess the potency and efficiency of conversion of the pre-mRNA to the desired splice form and the level of production of the desired protein. Of course, for allele-selective ASOs, splicing ASOs, and any other ASO mechanism, the standard selectivity and safety parameters are studied as well. In addition, the potential of the ASO for hybridization-based off-target effects is evaluated *in silico*. The effects of the ASO on any transcripts that are expressed in the cells of interest that have a meaningful probability of hybridizing with the ASO are then evaluated in patient-derived cells. Next, we assess whether the entire cellular phenotype is corrected by the ASO. Attractive ASOs are then evaluated for safety and tolerability in a number of specialized *in vitro* systems (discussed below) and rodent toxicology studies. At this juncture, a detailed formal presentation is made to the Research Management Committee (RMC), a committee of ASO experts and specialists in genetic diseases and clinical studies. In aggregate, members of this committee have hundreds of years of experience with ASOs and several have worked in the ASO field since the first companies were founded to pursue the creation of the technology in 1988/1989. The RMC then submits its recommendation to the n-Lorem executive committee, which makes the final decision to advance an ASO to IND submission.

### Establishing a clinical trial plan and protocol

During the preclinical development of the ASO and prior to submitting an IND to the FDA, we collaborate with the submitting physician to develop the clinical plan, which includes treatment goals and prespecified clinical outcome measures to be assessed. The clinical plan is then presented to a committee comprised of ASO and clinical trial experts, the Study Treatment and Review (STAR) committee (Fig. 1). The IND is then submitted to the appropriate division of the FDA. The protocol and informed consent are reviewed by the Institutional Review Board (IRB) of the treating institution. Lastly, the overall clinical performance, with particular focus on the safety and tolerability of the entire portfolio of clinically administered ASOs, is reviewed quarterly by the Data Safety Monitoring Board (DSMB). We consider every one of these “in process” quality assurance steps to be critical to providing optimal ASO treatment to appropriate patients.

### Modified cross-over clinical trial design

Historically, single-patient clinical trials have employed a cross-over design in which a patient is treated with placebo or a reference agent, and then administered the experimental agent or vice-versa [32]. However, given the long duration of ASO effects and the severity of the diseases that most nano-rare patients present, we use a modified cross-over design. During the time it takes to discover and develop an experimental ASO for a patient, jointly with the treating physician, we select primary, secondary, and exploratory treatment goals and the standard clinical measures to be used to assess the activity of the ASO. We then collect baseline data for 6 months or longer (depending on how long it takes to initiate treatment) and compare pretreatment to on-treatment assessment of the agreed endpoints. This approach has effectively provided high quality data that quantitate benefit and potential ASO AEs [33].

### Dose ranges, frequency, and dose escalation schemes

For IT-dosed ASOs, in large clinical trials, PS 2′-MOE ASOs have been dosed as high as 120 mg every 8 weeks [34]. Given that experience with clinical use of PS ASOs developed under the nano-rare patient ASO guidance is limited, we have chosen to dose very conservatively. We typically, then, initiate dosing at 20 mg a month for 3 months (to more rapidly achieve steady state CSF concentrations that are in the therapeutic range), and then dose quarterly. The quarterly dose is escalated from 20 to 50 mg. We then typically treat at 50 mg quarterly for three doses, then escalate to 100 mg maximum quarterly dosing. As we gain experience, we may increase the starting dose, escalation schedule, and maximum dose.

For IVT-dosed PS 2′-MOE ASOs, dosing is typically initiated at 60 μg in each eye quarterly for three doses, and then every 6 months. Doses are escalated from 60 μg to120 μg, and then 200 μg. For SC-dosed GalNAc PS 2′-MOE ASOs, the initial dose is typically 40 mg monthly. Doses are escalated to 80 mg, and then, if necessary,100 mg monthly.

### The n-Lorem nano-rare patient research and treatment network

**Figure 4.**
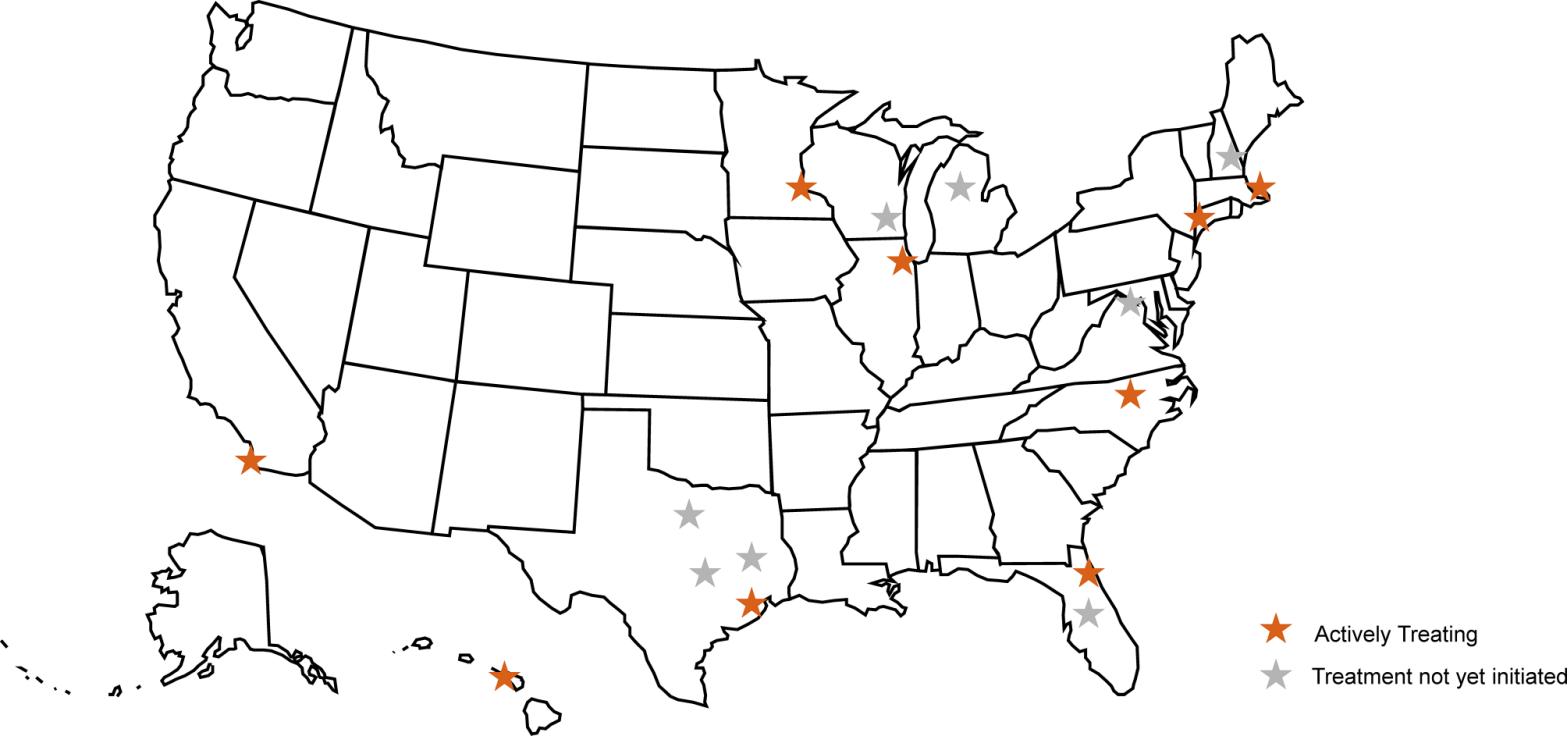
The n-Lorem nano-rare treatment and research network.

Though we have made significant progress in establishing a pan-USA network of outstanding clinical investigators experienced in the diagnosis and management of genetic diseases (Fig.4), we continue to seek additional sites as it is often difficult for patients and families to travel.

### Demand and demographics

**Figure 5.**
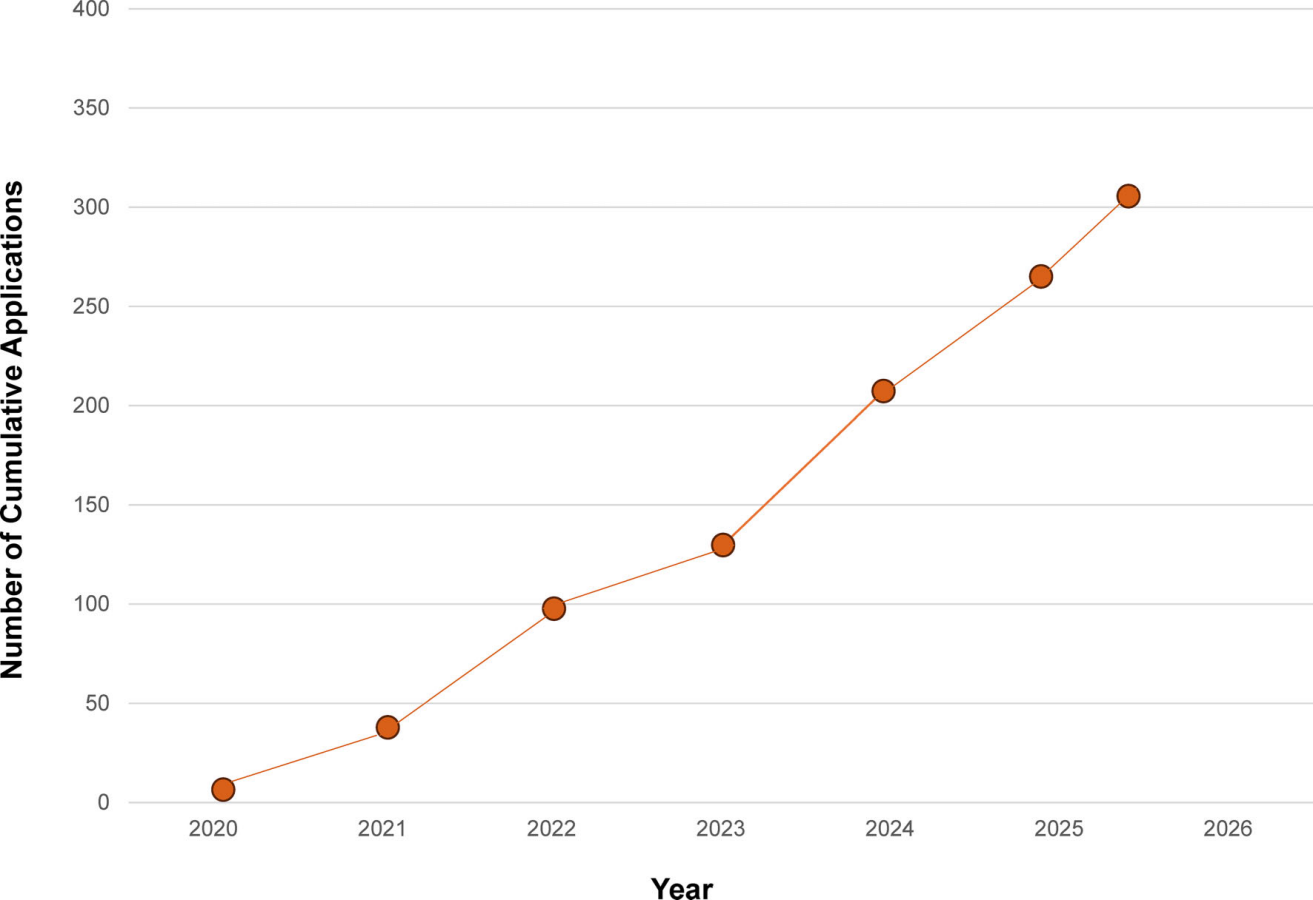
Cumulative applications by year.

**Figure 6.**
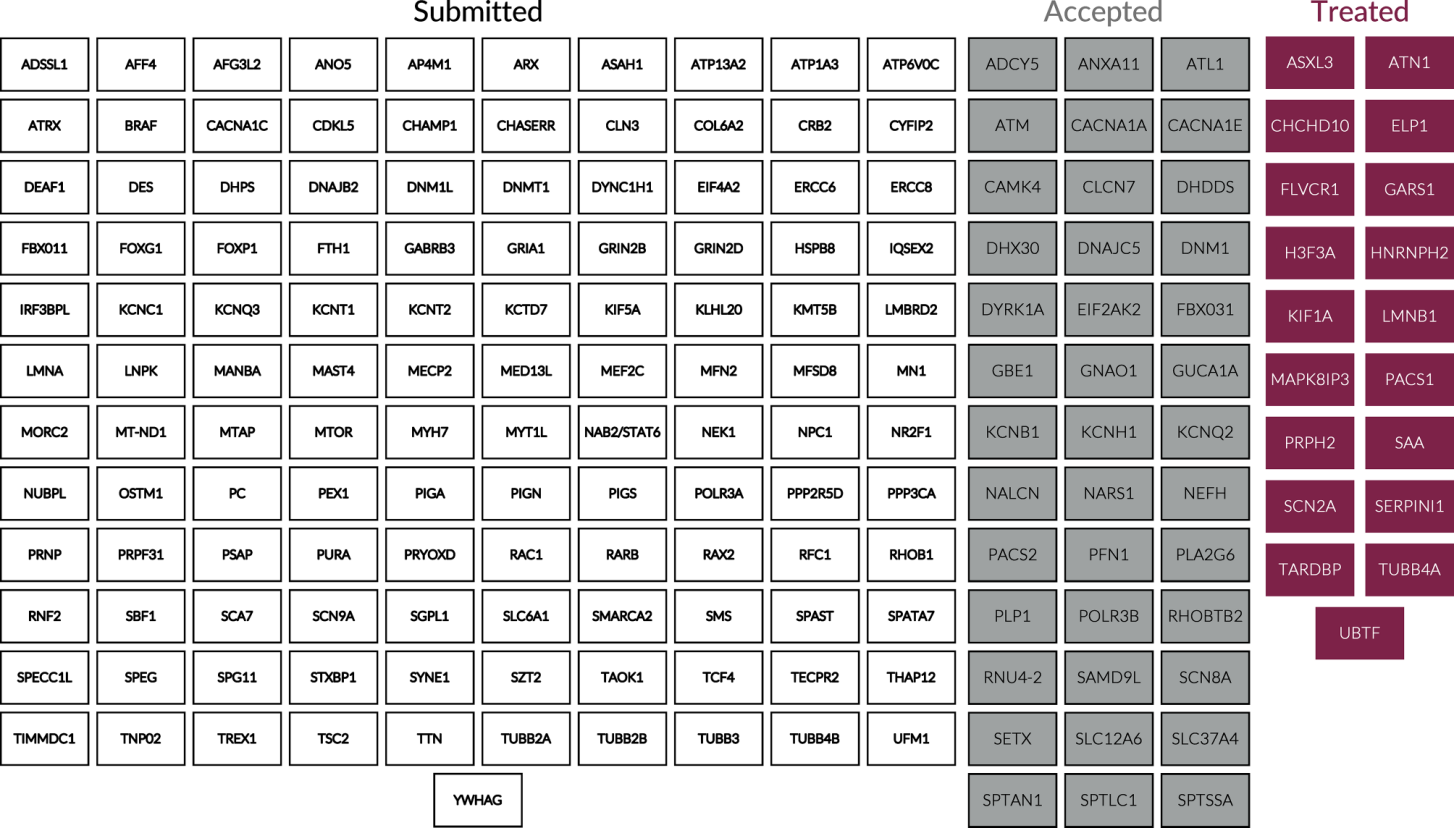
Summary of affected genes submitted. Submitted applications (white boxes), accepted applications (gray boxes), and genes of patients currently treated (burgundy boxes).

**Figure 7.**
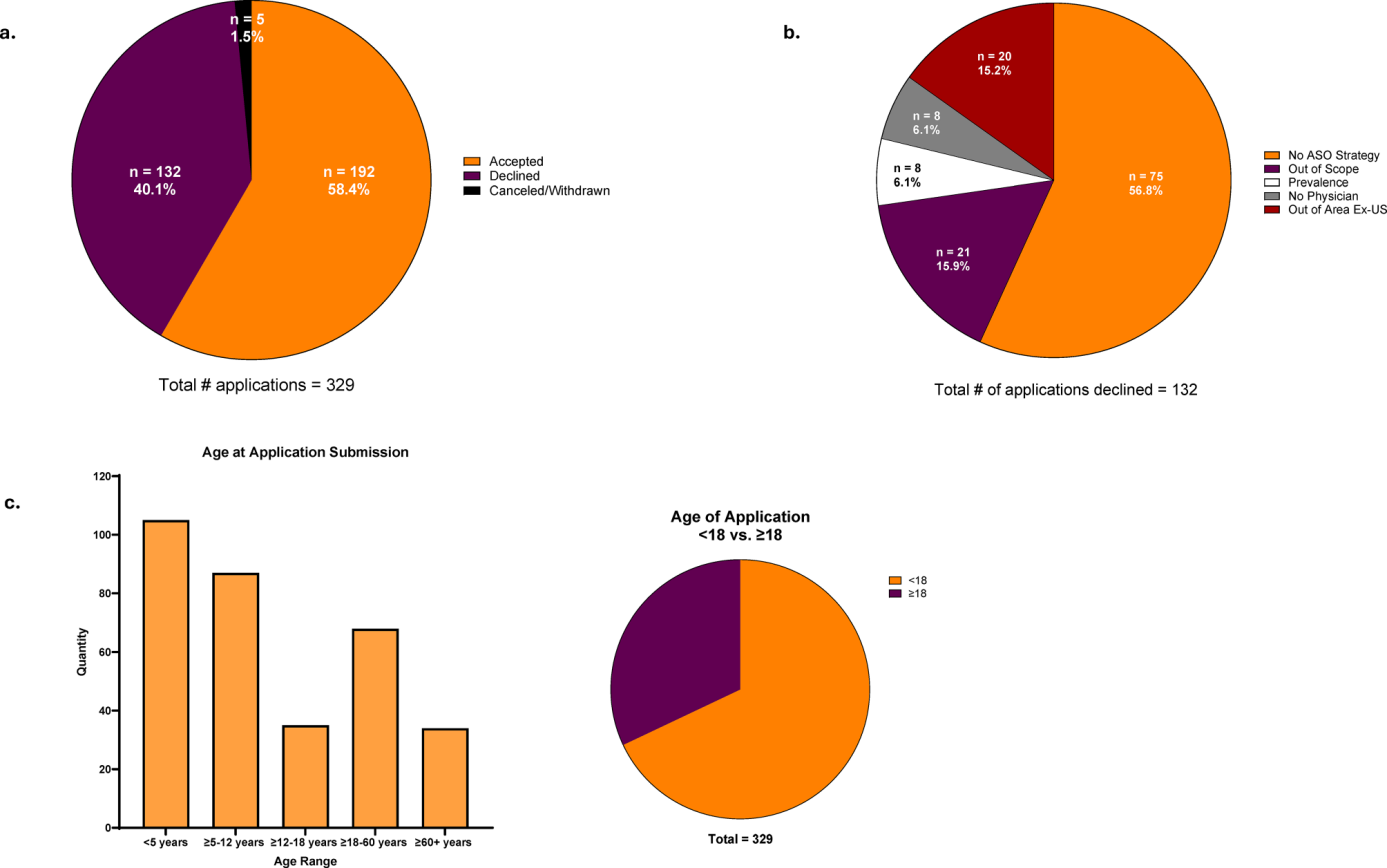
Patient demographics and ASO characteristics. (**a**) Fraction of applications accepted (orange), declined (burgundy), or withdrawn (black). (**b**) Reasons for rejection of applications (orange: no ASO strategy, burgundy: out of scope, white: too prevalent, gray: no physician, and red: ex-US). (**c**) Ages of patients who applied for treatment by age group (left panel); applications for patients under 18 (orange), and over 18 years of age (burgundy) (right panel).

Demand has greatly exceeded initial expectations (Fig. 5), necessitating much more rapid growth than planned. Though we accept patients with diseases expressed in the eye, liver, and kidneys, neurological diseases comprise the bulk of submissions. 184 of 192 applications accepted for treatment have been for patients with mutations that result primarily in CNS manifestations. While it may be the case that pathogenic nano-rare mutations that result in severe diseases are more prevalent in the CNS than other organs, we suspect that the disparity in applications may also be secondary to a more robust response of neurologists than other specialists to the opportunity presented by n-Lorem and potentially the greater need for treatment of CNS disorders. As of July 31, 2025, we received nearly 400 applications and completed initial processing (determined whether the application could be accepted for potential treatment or not) for 329 applications. The apparent increase in the fraction of applications accepted (Fig. 5, dashed line) is an artifact of a slight change in our process. In 2023, we implemented an additional triage step by establishing a pre-application committee to identify incomplete or inappropriate applications and this resulted in the apparent increase of applications accepted. As expected, diverse genes are affected by nano-rare mutations (Fig. 6); ~58% of the 329 applications for which the initial review has been completed was accepted for treatment (Fig. 7a). The main reasons for rejection of applications for treatment were that mutations were not considered amenable to current ASO treatment, diseases were expressed in organs that we are not treating at present, or an inadequate understanding of the phenotype and progression rate (Fig. 7b). As clinical investigators gain experience, we anticipate that the fraction of applications that require additional work to enable a treatment decision will decline.

Our patients are not only children. In fact, ~30% of the applications received were for patients >18 years old (Fig. 7c). Approximately 64% of our patients had heterozygous TGOF mutations in essential genes and required allele-selective RNase H1 ASOs. Twenty-seven percent could be treated with non-allele-selective RNase H1-activating PS ASOs. Nine percent of patients required PS ASOs designed to alter splicing and 1% (1 patient) required PS ASOs designed to increase the translation of a specific protein. Other relevant demographic characteristics are summarized in Fig. 7. A meaningful change in demographics over the first 5+ years of operation is that the fraction of applications for genes that were the subject of earlier applications has increased. The fraction of applications received that expressed mutations in the same gene as a previous patient in 2020 was 27%, and in 2024 and by July 31, 2025, 74 and 77%, respectively. As most applications have been to treat mutations with CNS manifestations, the increasing fraction of genes that were the subject of previously submitted applications suggests that we may have a reasonable representation of the types of genes involved in nano-rare diseases with CNS manifestations. Further, the fraction of applications for patients expressing the same pathogenic variant has increased from 21% in 2020 to 57% in 2024. This shift has important implications with regard to cost per patient as the cost to use a pre-existing ASO to treat new patients is a small fraction of the cost of discovering and developing a new ASO. We attribute a significant fraction of this shift to very active patient groups and investigators responding to the availability of potential treatments. As a result of these trends, the number of ASOs being used to treat multiple patients has increased significantly (Table 2).

**Table 2.** ASOs used to treat more than one patient

ASO	Gene	ASO Strategy	Patients Treated
nL-KIF1-001	KIF1A	RNase1 (Allele-selective)	2
nL-TARD-001	TARDBP	RNase1 (Allele-selective)	2
nL-TUBB4-001	TUBB4A	RNase1 (Non-allele-selective)	1
nL-ATN1-002	ATN1	RNase1 (Non-allele-selective)	2
nL-CHCHD10	CHCHD10	RNase1 (Non-allele-selective)	9
nL-RNPH2-001	HNRNPH2	RNase1 (Non-allele-selective)	3
nL-IKBK-001	ELP1	Splicing	2

Kinesin family member 1A (KIF1A), TAR DNA-binding protein (TARDBP), tubulin beta-4A (TUBB4A), atrophin 1 (ATN1), coiled-coil-helix-coiled-coil-helix domain-containing protein 10 (CHCHD10), heterogeneous nuclear ribonucleoprotein H2 (HNRNPH2), phosphofurin acidic cluster sorting protein 1 (PACS1), elongator acetyltransferase complex subunit 1 (ELP1).

### The n-Lorem pipeline

**Figure 8.**
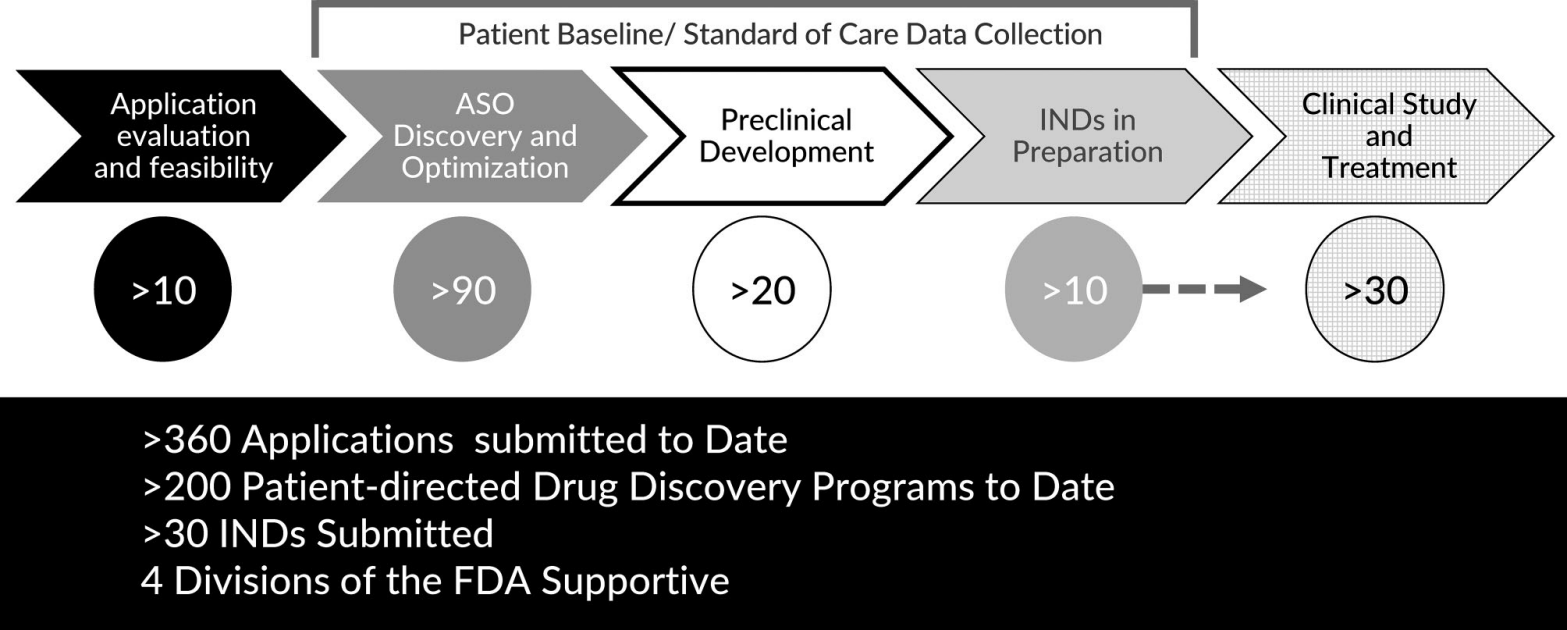
The n-Lorem pipeline.

The n-Lorem pipeline is shown in Fig. 8. Our goal is to process all applications and provide a response to the submitting investigator within 6 weeks of receipt of the application. Excluding applications that required additional work to determine eligibility for treatment, the average time from submission of an application to a decision was 74 days. Though our goal is to initiate dosing of patients within 18–24 months of acceptance of an application, since the demand exceeded available funding, the average time to treatment to first dose was >2 years.

To date, we have filed US INDs to the two neurological divisions, the cardio-renal and the ophthalmological and the rare disease divisions of the FDA, and one Clinical Trial Application (equivalent to a US IND) to Health Canada, vastly exceeding our original plans. Though the reviews by the FDA have been thorough and we have had to respond to numerous queries, all five of the divisions with which we have interacted have responded constructively and rapidly in accord with the guidance. We have had no INDs rejected.

### Clinical safety and tolerability

#### Typical safety assessments

After administration of PS 2′-MOE ASOs, any symptoms suggestive of an AE are evaluated and all standard safety assessments are performed. For ASOs delivered IVT, additional safety is evaluated by the treating ophthalmologist at each clinical visit. The assessments typically include evaluation for inflammation, cataract development, effects on visual fields, and acuity. Electroretinograms are also typically performed. For IT-administered PS 2′-MOE ASOs, additional studies can include CSF opening pressure and CSF cellularity, and protein content are routinely monitored and MRIs are conducted if indicated [31].

**Figure 9.**
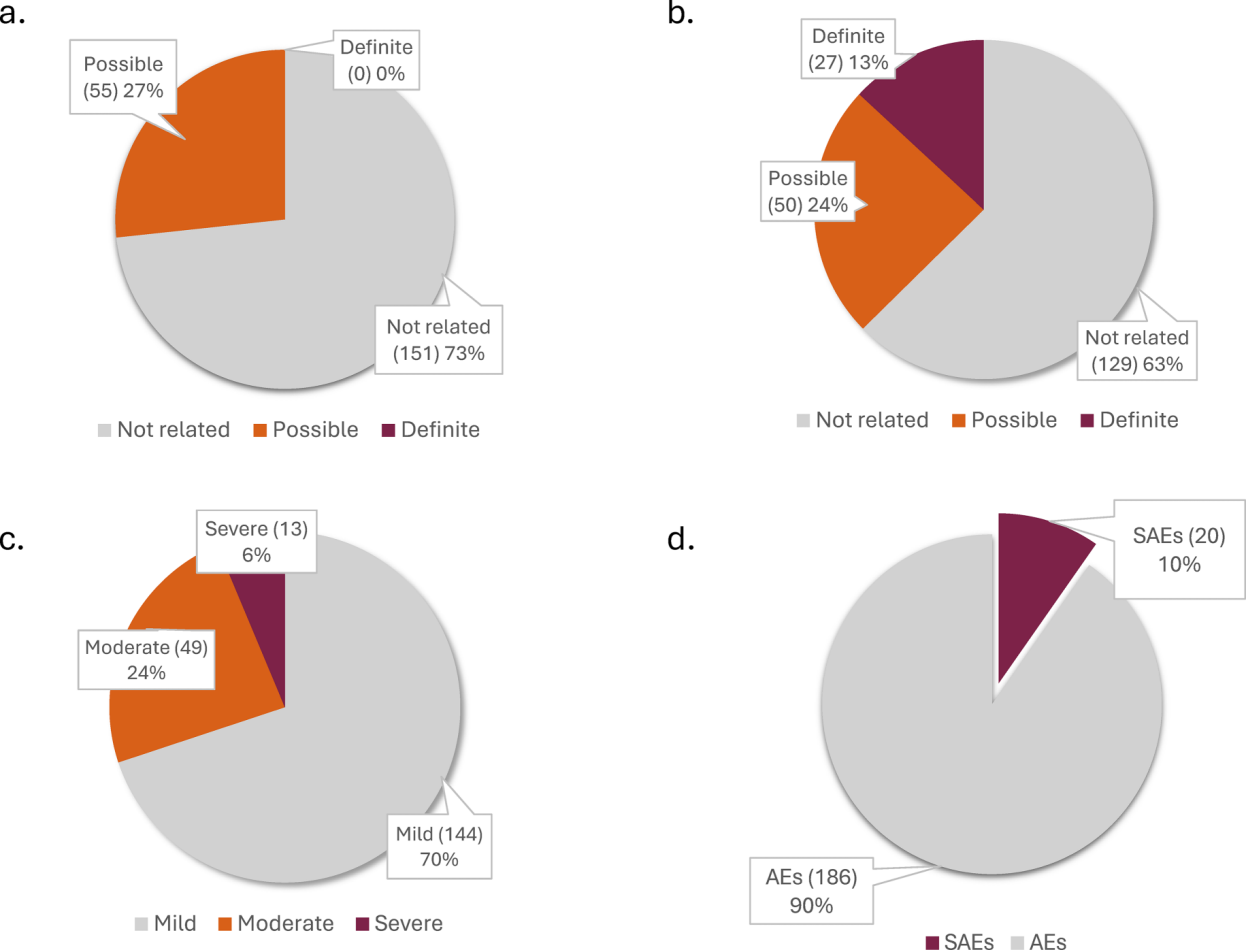
Safety and tolerability of clinically administered PS ASOs: (**a**) AE relatedness to administration of study drug: not related (gray) and possibly related (orange). (**b**) AE relatedness to drug administration: not related (gray), possibly related (orange), and definitely related (burgundy). (**c**) Severity of AEs: mild (gray), moderate (orange), and severe (burgundy). (**d**) Distribution of reported AEs: non-serious AEs (gray) and SAEs (burgundy).

Safety data are reviewed quarterly by the DSMB, comprised of external experts in ASO technology and clinical trials of novel experimental medicines. A full manuscript describing the safety and tolerability of n-Lorem PS ASOs as of January 2023 has been reported [19] and an updated analysis of safety and tolerability has been submitted for publication. Therefore, in this perspective, we simply provide an overview of our experience to date. As of July 31, 2025, our safety database (Table 3) was comprised of data from >32 patients, most of whom express pathogenic mutations that alter CNS functions and receive monthly IT dosing for the first three doses, and then quarterly dosing. One patient with retinal manifestations has been treated IVT quarterly initially, then every 6 months and one patient with serum amyloid1 (SAA-1)-related renal amyloidosis has been treated subcutaneously (SC) monthly. The total number of doses administered is >180, >10 patients have been treated for >1 year with one patient approaching 3 years of treatment. As expected, numerous AEs have been reported across patients with rare diseases (Fig. 9). Of AEs associated with ASO administration, 37% were considered possibly or definitely related by the investigators (Fig. 9b) and essentially all of the administration related AEs occurred in patients who were treated IT. All the drug administration-related AEs were consistent with expectations for IT administration and were comprised of headaches or pain at the site of lumbar puncture. Seventy percent of all reported AEs were considered “mild” in severity (Fig. 9c). Across all reported events, 10% were serious adverse events (SAEs) (Fig. 9d). No ASO-related SAEs have been reported and of the AEs reported, 27% have been considered possibly-ASO related (Fig. 9a).

**Table 3.** The safety database

Number of INDs	>35
Number of patients treated	>35
Total patient years of treatment	>30 years
Number of ASO doses administered	>180
Maximum duration of treatment	>2.5 years

One ASO-related AE has been identified in a female patient with a heterozygous TGOF mutation in kinesin family member 1A (KIF1A), an isoform of kinesin that acts as a motor to enable endosomes to move along microtubules. The mutation results in severe, progressive neurodegeneration. This patient [34] was severely affected at initiation of treatment and continues to demonstrate significant benefit in all affected domains and has been treated for almost 3 years (see below). However, over the past 12 months, the patient has experienced transient mild fevers occurring shortly after IT dosing. These symptoms recurred after each dose despite pretreatment with corticosteroids and discontinuation of the anesthetic, propofol, used to facilitate IT dosing. To our knowledge, this AE has not been observed with any other IT-administered PS 2′-MOE ASO. Consequently, we have reported this to the FDA and amended informed consents. Because the patient continues to demonstrate remarkable benefit, we are continuing to treat at a lower dose while carefully monitoring CSF pressure, CSF complement split products, and cytokines. A manuscript that describes this AE in detail is being prepared.

#### Clinical benefit

Though we are confident that the modified cross-over clinical trial design is providing high quality reasonably quantitative data [31], we prefer the term, benefit, rather than efficacy as efficacy has been associated with results obtained in randomized, well-controlled clinical trials in groups of patients with a shared diagnosis. As of July 31, 2025, 22 patients have been treated for >6 months and are evaluable for analysis of benefit. All 13 of the non-ALS patients have met protocol-specified endpoints for benefit. In fact, in most evaluable patients, the benefit observed has been substantial [19, 31]. Details of all patients have or will be published as clinical case reports. In one patient with a mutation in the *SAA-1* gene that results in misfolding and precipitation in kidneys, prior to initiating treatment the glomerular filtration rate (GFR) had been declining by 5–7 ml/min/m^2^ per year and the patient experienced significant proteinuria. After 6 months of treatment, SAA levels were significantly reduced, and eGFR has been stabilized for >12 months.

Similarly, a single patient with a genetically caused retinal disorder has been treated for sufficient time (>1 year) to assess benefit. This patient expresses a missense mutation in exon 8 of *FLVCR-1 *(feline leukemia virus choline receptor subgroup-related protein 1) gene that results in a novel 3′ splice site that causes defective splicing, thereby inactivating the protein. As FLVCR-1 protein is a choline transporter, clearance of iron from the cytoplasm of neuronal and retinal cells is reduced. The syndrome includes severe ataxia, loss of proprioception, and insensitivity to pain, as well as progressive vision loss. Because the patient’s vision loss was progressing rapidly and blindness would exacerbate the issues associated with ataxia, loss of proprioception, and pain insensitivity, the choice was made to attempt to delay or prevent further vision loss by treating IVT. No ASO-related AEs have been observed, and visual acuity has been stable for more than a year.

As of July 31, 2025, 9 of 11 ALS patients with a pathogenic variant in Coiled-Coil-Helix-Coiled-Coil-Helix Domain-Containing Protein 10 (CHCHD10) associated ALS are partially evaluable for benefit. All nine patients have experienced declines in neurofilament light chain, however, given the naturally slow rate of progression of CHCHD10-ALS, more evaluation time will be required to determine whether this biomarker change is associated with functional stabilization or improvement. One patient who had quite advanced disease ALS [ALS-FRS of 16 (of 48) at initiation of treatment] succumbed to an RSV (respiratory syncytial virus) pneumonia, unrelated to treatment.

Table 4 presents the genotypic and phenotypic characteristics of the patients with non-ALS CNS diseases as well as the prespecified treatment goals and clinical measures and summaries of the benefits observed. The patients display mutations in genes that encode several functional classes of proteins that are associated with an array of manifestations.

**Table 4.** Summary of evidence of benefit for patients with neurological diseases other than ALS

Patient ID	Gene and mutation type	ASO strategy	Route	Dose frequency	Max dose to date	Phenotype at Tx start	Relevant treatment goals	Relevant study endpoint	Summary of clinical benefitfor relevant endpoints
nL00255	KIF1A - missense mutation	ASRNase H1 ASO	IT	Every 3 months	100 mg, current: 80 mg	Medically refractory epilepsy requiring rescue medication Primarily non-ambulatory; able to take supported steps Marked tremor Nonverbal Severe neurodevelopmental impairment Severe peripheral neuropathy in extremities	Reduce seizure burdenImprove: – motor function – fall frequency – quality of life	Seizure frequency Motor function assessments Frequency of falls Cognitive assessment Quality-of-life questionnaire	Reduced seizure frequency and severity Reduced tremor severity Reduced neuropathic pain Fewer falls with better balance (now non-ambulatory following ankle sprain) Improved expressive communication Improved attention and awareness Increased stamina Increased independence with improved quality of life
nL00037	KIF1A - missense mutation	ASRNase H1 ASO	IT	Every 3 months	80 mg	Mixed tone abnormalities (spasticity, hypotonia, tremor) Non-ambulatory Nonverbal Global developmental delay with progressive divergence from age-appropriate milestones	Delay seizure onsetImprove: – motor skills and mobility – communication – sleep	Motor function assessments and tracker Communication assessments (BSID4, ORCA, Vineland 3) Sleep tracker	Improved expressive verbal communication Improved cognitive function Reduced tremor severity Reduced muscle contraction with better ability to crawl
nL00333	SCN2A - single base substitution	ASRNase H1 ASO	IT	Every 2–3 months	80 mg, current: 40 mg	Medically refractory epilepsy, requiring multiple antiseizure medications including high-dose phenytoin, and rescue therapy Nonverbal, uses assistive communication device Severe intellectual disability Autism spectrum disorder	Reduce seizure frequency and durationImprove: – communication and language – cognitive and adaptive functioning – sensory dysfunction – irritability and aberrant behavior	Seizure frequency and duration Language and communication (ORCA) Cognitive/adaptive function (Vineland 3) Irritability and aberrant behavior (ABC) Sensory dysfunction (Short Sensory Profile)	Reduced seizure frequency and severity Reduced antiseizure medication burden, d/c high dose Phenytoin No ER visit for rescue medications Improved stereotypic behavior and irritability Improved receptive communication Reduction in some aberrant sensory seeking behaviors
nL00001	SCN2A - single base substitution	ASRNase H1 ASO	IT	Every 2–3 months	40 mg, current: 30 mg	Medically refractory epilepsy with multiple seizure types (myoclonic, focal, tonic) Non-ambulatory Hyperkinetic movement disorder with choreoathetosis and dyskinesia Nonverbal, uses assistive communication device Severe global neurodevelopmental delay Chronic gastrointestinal dysfunction	Reduce seizure burdenImprove: – motor function and movement disorder – communication – global neurodevelopment – gastrointestinal function	Seizure frequency Motor function and movement assessments Communication (ORCA) Neurodevelopment /behavior (BSID4, Vineland 3, ABC) GI function (Bristol Stool Scale)	Reduced seizure frequency and severity Improved mobility Improved communication Improved gastrointestinal symptoms Reduced irritability Improved social interactions
nL01183	ATN1-trinucleotide repeat expansion	NASRNase H1 ASO	IT	Every 3 months	80 mg	Epilepsy Marked cerebellar ataxia with very limited ambulation with support Dysarthric speech Intellectual disability with global developmental delay Anxiety disorder, attention-deficit/hyperactivity disorder	Reduce seizure frequency, duration, and antiseizure medication burdenImprove: – ataxia and mobility – communication – cognition and intellectual functioning – quality of life	Seizure frequency, duration, and medication use (seizure diary) Mobility and ataxia (accelerometer, video analysis) Communication (ORCA) and cognition assessments Quality-of-life questionnaire	Improved mobility, including independent ambulation Improved motor coordination Increased functional independence Improved quality of life
nL98087	hnRNPH2 -missense mutation	NASRNase H1 ASO	IT	Every 3 month	50 mg	Epilepsy with tonic-clonic and atypical seizure types Generalized hypotonia Non-ambulatory; severe motor impairment Severe expressive communication impairment with absent speech Intellectual disability with developmental delay and regression Behavioral dysregulation Chronic gastrointestinal dysfunction with feeding difficulties	Reduce seizure frequency Improve: – gross and fine motor function – communication – cognitive function – maladaptive behavior and quality of life	Seizure frequency Gross and fine motor assessments Dystonia assessment Communication and cognition (BSID4, Vineland 3, ORCA) Behavioral assessments	Improved awareness Improved social interaction Improved muscle control
nL92722						Absence epilepsy Non-ambulatory; severe motor impairment with generalized hypotonia Severe expressive communication impairment with absent speech Intellectual disability with developmental delay Behavioral dysregulation Chronic gastrointestinal dysfunction with feeding difficulties			First independent steps achieved after five doses of study drug Ability to climb stairs independently while holding railings Improved fine motor skills Improved communication Improved dystonia
nL22680						Epilepsy Generalized hypotonia Ambulatory Regression in communication with complete loss of previously acquired speech Global developmental delay Autism spectrum disorder and behavioral disorder			Improved fine motor skills, Increased independence in activities of daily living (e.g. tooth brushing) Reduced tremor Improved attention and hand–eye coordination enabling independent feeding
nL00298	H3F3A - missense mutation	NASRNase H1 ASO	IT	Every 3 months	50 mg	Cerebella ataxia with poor balance Hypotonia Nonverbal with absent expressive language and severely impaired receptive language Severe global developmental delay Sleep-wake dysregulation	Improve:– communication ability – cognition and adaptive behavior – motor coordination and balance – sleep regulation and quality of life	Communication (Weighted Intentional Communication Scale, BSID-4, Vineland 3, ORCA) Cognitive and adaptive behavior scales Motor/balance assessments Sleep questionnaires	Improved communication, Improved focus and alertness and motor planning/coordination
nL00214	TUBB4A-missense	NASRNase H1 ASO	IT	Every 3 months	50 mg	Tone abnormalities (spasticity) Non-ambulatory Nonverbal Global developmental delay Severe feeding difficulties	Improve:– gross motor function – feeding skills and nutritional status	Motor milestones Spasticity (Tardieu Spasticity Scale) Feeding assessments	Reduced wrist spasticity New gross motor milestones achieved, including head control, supported sitting, and active lower-extremity movements Increased exploratory behavior and mobility (commando crawling) Increased appetite
nL00808	ELP1-exon skipping	Splicing ASO	IT	Every 4 months	24 mg	Mild gait ataxia Ambulatory without assistance Decreased pain and temperature sensation Decreased visual acuity Dysphagia Gastrostomy tube dependence nutrition	Improve:– sensory perception (temperature, vibration, proprioception)– gait and balance– peripheral nerve function	Quantitative Sensory Testing (temperature and vibration) Mobility and gait analysis Nerve conduction studies	Improved gait stability with reduced sway during straight-line ambulation Improved sensory processing, including enhanced temperature discrimination Evidence of peripheral nerve fiber regeneration on knee / ankle skin biopsy Increased ELP1 protein levels

AS: allele-selective;

NAS: nonallele-selective;

IT: intrathecal;

ORCA: observer rated communication ability;

BSID-4: Bayley Scales of Infant and Toddler Development 4th edition;

ABC: aberrant behavior scale.

#### Limitations

Though ASO technology is versatile, true null mutations cannot be addressed with this technology unless there is a paralog that can be increased or altered to compensate for the functions lost due to the null mutation. Nor can we use ASOs to address genomic deletions. However, many LOF mutations can potentially be addressed by altering RNA metabolism and processing or upregulation of translation [35, 36]. We also limit our efforts to hepatic and renal targets after SC administration, retinal mutations treated IVT and CNS diseases treated IT because we have detailed understanding of the suborgan PK/PD of PS ASOs of several chemical classes in those organs [37, 32]. Lastly, largely because of resource limitations, with the exception of one patient in Canada, we are currently treating only patients located in the USA.

#### Conclusions and future perspectives

Our experience provides important added insights into the diagnosis and management of extremely rare genetic diseases. We believe that the vast majority of patients with pathogenic nano-rare mutations are never diagnosed and we have reported that time from symptom onset to diagnosis is almost 5 years with the time to diagnosis ranging from 1 month to 36 years [19]. These data demonstrate the critical need to introduce whole genome sequencing into newborn screening and diagnosis. Only when all humans are sequenced at birth will the true prevalence of each extremely rare mutation be known and only then will it be possible to intervene early in the pathological process.

The number of applications for treatment received speaks to the scale of the challenge of extremely rare genetic diseases. Though each mutation may affect only a few humans, the total prevalence of extremely rare genetic diseases is quite large as demonstrated by our experience. The costs to healthcare systems around the world of care of such patients is significant, suggesting that non-profit models are unlikely to be sufficient to address the total population of extremely rare genetic diseases. New models may be needed in which the studies required for regulatory approval are feasible and encourage commercial development of novel medicines. New approaches to pricing novel medicines for extremely rare diseases may also be required if all patients are to have access to therapies.

We have previously reported that nano-rare mutations, like other pathogenic mutations, can result in divergent phenotypes [19]. While this is not surprising, as we gain experience, our data and the thoroughness of genotypic and phenotypic analyses should provide a tool of increasing value for clinicians who care for patients with nano-rare mutation-caused diseases.

Importantly, the safety and tolerability profiles of the ASOs discovered and developed by n-Lorem and administered clinically suggest that the guidance issued by the FDA provides sufficient protection against AEs if ASOs are discovered and developed by organizations with the requisite experience and rigorous ASO discovery and development processes, particularly when coupled to cautious dose escalation schemes. Our experience also argues that an industrialized approach, such as that established at n-Lorem, is an effective means to assure quality judgements about treatment decisions. Our patient with a *KIF1A* mutation and treated for almost 3 years has experienced an ASO-related AE that seems to be novel and concerning. The precise mechanism accounting for the AE is not understood. Another patient with a TGOF mutation in *KIF1A* and amenable to treatment with the same ASO has, to date, experienced no ASO-related AEs. However, this patient is younger and less severely affected and has been treated for less time than the patient who has experienced the ASO-related fever.

The versatility of ASO technology is demonstrated by the organs treated, routes of delivery employed, and the mechanistic classes of ASOs used. Key advantages include efficiency, rapidity, cost effectiveness of drug discovery, and the ability to predict basic PK/PD properties of an experimental ASO based on previously tested ASOs of the same chemical class.

Given the experience with PS 2′-MOE ASOs [5–8], it is not surprising that an ASO administered IVT met prespecified benefit endpoints in a patient with retinal disease or that an SC-administered ASO stabilized renal function in a patient with SAA amyloid kidney disease. Similarly, prior experience in ALS might have predicted success in other genetically caused types of ALS as we have observed in *CHCHD10* patients. However, we also saw significant unexpected benefits in our patients. The impressive reductions in seizure activity observed in several patients with mutations in different genes are notable. Similarly, the reduction in neuropathic pain observed in a *KIF1A* patient and the improvement in autonomic dysfunction noted in a few patients as well as improvement in autistic symptoms in several patients are important new observations for the technology.

The recovery of functions previously lost by the patients with pathogenic mutations in *KIF1A*, Atrophin-1 (*ATN1*), the sodium voltage-gated channel alpha subunit 2 (*SCN2A*), and other genes plus the acquisition of new skills such as walking independently, catching a ball, and other skills in our patients may encourage more optimism about recovery or acquisition of developmental milestones. Our experience demonstrates that these patients are unique “experiments of nature” and can provide important insights into health and disease.

#### Challenges remaining

The single greatest challenge to responding to the needs of the nano-rare patient is the scale of the demand. The number of applications for treatment has been much larger than expected, requiring more rapid growth than planned and the recruitment of vastly more funding than expected. In fact, our industrialized systems are readily scalable and could meet the demand, but the recruitment of funds has not kept pace with the demand. This means that today there is a growing number of patients for whom treatment is delayed. In response, we have more rapidly implemented partnerships with commercial organizations as a means of supplementing the donations we receive. Longer-term, some form of commercial approval in which the scale of regulatory demands for approval is matched to scale of the patient population for an individual mutation, may be essential. To be affordable, novel commercial models may be needed.

Certainly, a single small non-profit like the n-Lorem Foundation cannot solve the scale problem, but our demonstration of the benefits of ASO treatment of patients appropriate to treat with ASOs may provide the proof of principle needed to encourage implementation of more holistic solutions. We have benefitted from the commitment of the FDA to address the needs of nano-rare patients, and we believe that the existing guidance can serve as a basis to establish a path to commercial approval. However, for the entire nano-rare patient population to have access to effective treatments, new approaches to pricing may be required [33]. Further, to economically justify the investment in the diagnosis and treatment of nano-rare patients, better data on the costs of care for untreated nano-rare patients are needed for comparison. Certainly, our experience suggests that the economic impact of nano-rare diseases is quite significant and extends well beyond the direct costs of care for patients because in most cases, at least one wage-earner must stop working to care for these severely affected patients.

Addressing the needs of nano-rare patients around the world is the second major challenge. Though we have been unable to expand beyond the US because of funding limitations, the challenges are more complex than simply funding. To date, no country other than the US has established clear regulatory guidance that would facilitate advancing ASO treatments of nano-rare patients in other countries. We are hopeful that the FDA’s pioneering guidance for ASOs for nano-rare patients can serve as a model for other regulatory agencies.

The limitations of antisense technology is a third major challenge. LOF mutations are the most frequent reason to reject an application for treatment. Obviously, true null mutations are not amenable to ASO treatment, but multiple mechanisms that increase the translation of specific proteins have been developed [35, 36, 38, 39] and efforts to enhance the performance of ASOs designed to increase levels of specific proteins may make it possible to treat many patients with LOF mutations. For true null mutations, gene replacement or gene editing may be the only viable approaches, but significant technological advances will need to be made before either approach is facile, cost-effective, and safe enough to be used on the scale required.

### Achieving a more holistic solution

To achieve a more holistic solution, we think that a path to commercial approval may be essential. Since the impediments to commercialization of ASO for nano-rare diseases are primarily economic, to be effective, solutions must address factors that result in unattractive returns on investments, particularly compared to investments in more prevalent disease indications. In that regard, the FDA recently took an important first step when it published draft guidance for commercialization of drugs for extremely rare diseases [40]. What may be particularly important about the draft guidance is that it builds on the current guidance for ASOs of well-understood chemical classes for nao-rare patients. The guidance for ASOs addresses two economically critical issues as it facilitates a rapid, relatively inexpensive path to acquiring clinical data in relevant patients. This rapid, cost-effective path to acquire clinical data supports access to data that can substantially reduce the risk of technical failure. Such data will support a significant reduction in the discount imposed on product opportunities at the time the decision to invest in developing a product candidate must be. Since the discount for risk of technical failure is very high, a significant reduction in that discount will enhance the imputed return on investment (ROI) for drugs for extremely rare diseases.

A second key component implied in the draft commercial guidance is a significant reduction in total cost of development, facilitated by a reduction in the demands for clinical data. Though not explicitly stated, if the guidance that is issued builds on the existing nano-rare IND guidance, it will reduce the demands for preclinical studies and facilitate rapid, relatively inexpensive acquisition of clinical data in the relevant patient population. The combination of enhanced probability of success and reduced cost of development should dramatically enhance the imputed ROI for investments in extremely rare indications. That said, our experience and advances in our understanding of traditionally defined diseases suggest that new approaches to Phase 3 studies and marketing may be needed. More than half of our patients express pathogenic mutations in genes that encode essential gene products and consequently require allele-selective ASOs. Since non-pathogenic SNVs are targeted to achieve allele-selectivity, for diseases like KIF1 A disease, several ASOs are required to treat the entire patient population. Since mounting multiple Phase 3 studies would be quite expensive, new Phase 3 designs that test several genetic medicines targeted to different non-pathogenic SNVs might be evaluated in a single Phase 3 study and this would reduce Phase 3 costs significantly. Such a Phase 3 study design has been proposed [41]. Moreover, recent progress in ALS suggests that many traditionally defined disease populations may be comprised of patients with pathogenic mutations in a number of genes [41], necessitating Phase 3 studies of several drugs that target correction of mutations in several different genes. Though a bit more complex, the cost of Phase 3 studies might be reduced via a similar composite design. Irrespective of the reason that several drugs may be required to treat an entire patient population, or the type of Phase 3 program, it seems likely that new approaches to marketing may be required as well. Rather than achieving an attractive ROI with a single “blockbuster” drug, commercial companies may need to become comfortable building a market by commercializing several drugs for a traditionally defined disease such as ALS.

In sum, the n-Lorem industrialized processes that assure appropriate patients are treated with optimal ASOs demonstrate that significant benefit in many patients may be achieved with an attractive safety and tolerability profile. Importantly, attention to the desperate needs of the nano-rare patient population has increased rapidly and there is significant evidence that innovative, broadly enabling, more efficient drug discovery platforms such as ASO technology, coupled to novel regulatory approaches and new commercial approaches, may result in more holistic solutions that address the needs of the entire nano-rare patient population in an affordable fashion.

To learn more about the important work n-Lorem is doing, please visit n-Lorem’s website (www.nlorem.org), and if interested in collaborating with n-Lorem please email info@nlorem.org.

## Supplementary Material

gkag504_Supplemental_Files

## Data Availability

Data underlying this article will be shared on reasonable request to the corresponding author.

## Appendix A: Investigators who submitted applications included in this manuscript

Abreu, Nicolas J. M.D.; NYU Langone Health

Acosta, Fernando M.D.; Cook Children’s Health Care System

Allen-Sharpley, Michelle M.D., Ph.D.; Cedars-Sinai Medical Center

Appu, Merveen M.D.; Valley Children’s Healthcare

Armstrong, Caren M.D., Ph.D.; UC Davis Health

Bain, Jennifer M.D., Ph.D.; Columbia University Irving Medical Center

Baker, Joshua D.O.; Lurie Children’s Hospital of Chicago

Batley, Kaitlin M.D.; UT Southwestern Medical Center

Berry-Kravis, Elizabeth M.D., Ph.D.; Rush University Medical Center

Bhoj, Elizabeth J. K. M.D., Ph.D.; Children’s Hospital of Philadelphia (CHOP)

Bonkowsky, Joshua Leitch M.D., Ph.D.; University of Utah Health

Borooah, Shyamanga MBBS, MRCP (UK), MRCSEd, FRCOphth, Ph.D.; UC San Diego Health

Brigham, Danielle M.D.; Columbia University Irving Medical Center

Calame, Daniel M.D., Ph.D.; Baylor College of Medicine

Chang, Irene M.D.; UCSF Benioff Children’s Hospital

Chao, Hsiao-Tuan M.D., Ph.D.; Baylor College of Medicine & Texas Children’s Hospital

Chung, Wendy M.D., Ph.D.; Boston Children’s Hospital & Harvard Medical School

Costain, Gregory M.D., Ph.D.; The Hospital for Sick Children & University of Toronto

Cousin, Margot Ph.D.; Mayo Clinic

Craigen, William M.D., Ph.D.; Baylor College of Medicine

Dagli, Aditi M.D., Orlando Health Arnold Palmer Hospital for Children

Dasouki, Majed M.D.; AdventHealth Medical Group

Demarest, Scott M.D.; Children’s Hospital Colorado & University of Colorado

Devinsky, Orrin M.D.; NYU Langone Health

Dowling, James M.D., Ph.D.; University of Pennsylvania, Perelman School of Medicine

Ebrahimi-Fakhari, Darius M.D., Ph.D.; Boston Children’s Hospital & Harvard Medical School

Eichler, Florian M.D.; Massachusetts General Hospital & Harvard Medical School

Evrony, Gilad M.D., Ph.D.; NYU Langone Health

Finkel, Richard M.D.; St. Jude Children’s Research Hospital

Friedman, Jennifer M.D.; Rady Children’s Institute for Genomic Medicine & University of California San Diego

Gertler, Tracy M.D., Ph.D.; Lurie Children’s Hospital of Chicago

Gissen, Paul MBCHB, Ph.D.; University College London

Goh, Li Meng Denise, MBBS; National University Hospital

Gonzalez-Duarte Briseno, Maria Alejandra M.D.; NYU Langone Health

Granadillo De Luque, Jorge Luis M.D.; Washington University in St. Louis, School of Medicine

Greally, John D.Med., Ph.D., F.A.C.M.G.; Albert Einstein College of Medicine

Grinspan, Zachary M.D.; Weill Cornell Medicine

Grunseich, Christopher M.D.; National Institute of Neurological Disorders and Stroke (NINDS)

Helbig, Ingo M.D.; Children’s Hospital of Philadelphia (CHOP)

Hirano, Michio M.D.; Columbia University Irving Medical Center

Ilieva, Hristelina M.D.; Thomas Jefferson University

Izumi, Kosuke M.D., Ph.D.; Children’s Hospital of Philadelphia (CHOP)

Jen, Joanna M.D., Ph.D.; Icahn School of Medicine at Mount Sinai

Julich, Kristina M.D.; Dell Medical School & The University of Texas at Austin

Kacpura, Abigail D.O.; Cleveland Clinic

Kaufmann, Horacio M.D.; NYU Langone Health

Khurana, Vikram M.D., Ph.D.; Massachusetts General Hospital

Kim-McManus, Olivia M.D.; Rady Children’s Hospital & University of California San Diego

Kimonis, Virginia M.D.; UC Irvine Health

Kruer, Michael M.D.; Phoenix Children’s Hospital & University of Arizona College of Medicine

Kuo, Sheng-Han M.D.; Columbia University Irving Medical Center

Lanpher, Brendan C. M.D.; Mayo Clinic

Le Pichon, Jen-Baptiste M.D., Ph.D., FAAP; Children’s Mercy Hospital

Leung, Nelson M.D.; Mayo Clinic

Liow, Kore M.D., FACP, FAAN; University of Hawai`i John Burns School of Medicine

McCourt, Emily M.D.; Children’s Hospital Colorado & University of Colorado

Mefford, Heather M.D., Ph.D.; St. Jude Children’s Research Hospital

Mencacci, Niccolò Emanuele M.D., Ph.D.; Northwestern Medicine

Mikati, Mohamad M.D.; Duke University School of Medicine

Moufawad El Achkar, Christelle M.D.; Boston Children’s Hospital & Harvard Medical School

Muriello, Michael M.D.; Medical College Wisconsin

Numis, Adam M.D.; UCSF Benioff Children’s Hospital

Olson, Heather M.D.; Boston Children’s Hospital & Harvard Medical School

Ortiz-Gonzalez, Xilma M.D., Ph.D.; Children’s Hospital of Philadelphia (CHOP)

Oskarsson, Bjorn M.D.; Mayo Clinic

Plecko, Barbara M.D.; Medical University of Graz

Poduri, Annapurna M.D., MPH; Boston Children’s Hospital & Harvard Medical School

Saenz, Margarita M.D.; Children’s Hospital Colorado

Sands, Tristan M.D., Ph.D.; Columbia University Irving Medical Center

Sanger, Terrence M.D.; Children’s Hospital of Orange County & UCI School of Medicine

Shahani, Dave N. M.D.; Cook Children’s Health Care System

Shillington, Amelle D.O.; Cincinnati Children’s Hospital

Shiloh-Malawsky, Yael M.D.; UNC School of Medicine

Shneider, Neil M.D., Ph.D.; Columbia University Irving Medical Center

Singh, Mandeep M.D., Ph.D.; Johns Hopkins University School of Medicine

Sorenson, Eric M.D.; Mayo Clinic

Sumner, Charlotte M.D.; Johns Hopkins University School of Medicine

Tekin, Mustafa M.D.; Miller School of Medicine, University of Miami

Thomas, Florian M.D., Ph.D.; Hackensack University Medical Center & Hackensack Meridian School of Medicine

Velinov, Milen M.D., Ph.D.; Rutgers Robert Wood Johnson School of Medicine

Vockley, Gerard M.D., Ph.D.; University of Pittsburgh & UPMC Children’s Hospital of Pittsburgh

Yu, Timothy M.D., Ph.D.; Boston Children’s Hospital & Harvard Medical School

Zaidman, Craig M.D.; Washington University in St. Louis, School of Medicine

Ziobro, Julie M.D., Ph.D.; University of Michigan Medical School

## References

[1] Crooke ST . A call to arms against ultra-rare diseases. Nat Biotechnol. 2021;39:671–7. 10.1038/s41587-021-00945-0. 34089038

[2] Borri A, Cerasa A, Tonin P et al. Characterizing fractal genetic variation in the human genome from the HapMap Project. Int J Neural Syst. 2022;32:2250028. 10.1142/S0129065722500289. 35579974

[3] Crooke ST . Meeting the needs of patients with ultrarare diseases. Trends Mol Med. 2022;28:87–96. 10.1016/j.molmed.2021.12.002. 35000835

[4] Crooke ST . Addressing the needs of patients with ultra-rare mutations one patient at a time: the n-Lorem approach. Nucleic Acid Ther. 2022;32:95–100. 10.1089/nat.2021.0046. 34520268

[5] Crooke ST, Liang XH, Baker BF et al. Antisense technology: a review. J Biol Chem. 2021;296:100416. 10.1016/j.jbc.2021.100416. 33600796 PMC8005817

[6] Crooke ST, Liang XH, Crooke RM et al. Antisense drug discovery and development technology considered in a pharmacological context. Biochem Pharmacol. 2021;189:114196. 10.1016/j.bcp.2020.114196. 32800852

[7] Vickers TA, Migawa MT, Seth PP et al. Interaction of ASOs with PC4 is highly influenced by the cellular environment and ASO chemistry. J Am Chem Soc. 2020; 142:9661–9674. 10.1021/jacs.0c01808. 32374993

[8] Crooke ST, Witztum JL, Bennett CF et al. RNA-targeted therapeutics. Cell Metab. 2019;29:501. 10.1016/j.cmet.2019.01.001. 30726759

[9] Crooke ST, Wang S, Vickers TA et al. Cellular uptake and trafficking of antisense oligonucleotides. Nat Biotechnol. 2017;35:230–7. 10.1038/nbt.3779. 28244996

[10] Crooke ST, Baker BF, Kwoh TJ et al. Integrated safety assessment of 2'-O-methoxyethyl chimeric antisense oligonucleotides in nonhuman primates and healthy human volunteers. Mol Ther. 2016;24:1771–82. 10.1038/mt.2016.136. 27357629 PMC5112040

[11] Crooke ST, Baker BF, Pham NC et al. The effects of 2'-O-methoxyethyl oligonucleotides on renal function in humans. Nucleic Acid Ther. 2018;28:10–22. 10.1089/nat.2017.0693. 29185862 PMC5790433

[12] Crooke ST, Baker BF, Witztum JL et al. The effects of 2'-O-methoxyethyl containing antisense oligonucleotides on platelets in human clinical trials. Nucleic Acid Ther. 2017;27:121–9. 10.1089/nat.2016.0650. 28145801 PMC5467133

[13] Crooke ST, Baker BF, Xia S et al. Integrated assessment of the clinical performance of GalNAc3-conjugated 2'-O-methoxyethyl chimeric antisense oligonucleotides: I. Human volunteer experience. Nucleic Acid Ther. 2019;29:16–32. 10.1089/nat.2018.0753. 30570431 PMC6386089

[14] U.S. Food and Drug Administration . Nonclinical Testing of Individualized Antisense Oligonucleotide Drug Products for Severely Debilitating or Life-Threatening Diseases Guidance for Sponsor-Investigators. https://www.fda.gov/regulatory-information/search-fda-guidance-documents/nonclinical-testing-individualized-antisense-oligonucleotide-drug-products-severely-debilitating-or. Date accessed 09 March, 2026.

[15] U.S. Food and Drug Administration . IND Submissions for Individualized Antisense Oligonucleotide Drug Products: administrative and Procedural Recommendations Guidance for Sponsor-Investigators. https://www.fda.gov/regulatory-information/search-fda-guidance-documents/ind-submissions-individualized-antisense-oligonucleotide-drug-products-administrative-and-procedural. Date accessed 09 March, 2026.

[16] U.S. Food and Drug Administration . IND Submissions for Individualized Antisense Oligonucleotide Drug Products for Severely Debilitating or Life-Threatening Diseases: clinical Recommendations. https://www.fda.gov/regulatory-information/search-fda-guidance-documents/ind-submissions-individualized-antisense-oligonucleotide-drug-products-severely-debilitating-or-life. Date accessed 09 March, 2026.

[17] U.S. Food and Drug Administration . Investigational New Drug Application Submissions for Individualized Antisense Oligonucleotide Drug Products for Severely Debilitating or Life-Threatening Diseases: chemistry, Manufacturing, and Controls Recommendations, Guidance for Sponsor-Investigators. https://www.fda.gov/regulatory-information/search-fda-guidance-documents/investigational-new-drug-application-submissions-individualized-antisense-oligonucleotide-drug. Date accessed 09 March, 2026.

[18] n-Lorem Foundation . https://www.nlorem.org/. Date accessed 09 March, 2026.

[19] Crooke ST, Cole T, Carroll JB et al. Genotypic and phenotypic analysis of 173 patients with extremely rare pathogenic mutations who applied for experimental antisense oligonucleotide treatment. medRxiv, 10.1101/2024.08.05.24310862, 20 October 2024, preprint: not peer reviewed..

[20] O’Rourke JG, Bachmann G, Mazur C et al. Acute neuronal inhibition response caused by phosphorothioate antisense oligonucleotides following local delivery to the central nervous system. Nucleic Acids Res. 2026;54:gkaf1333. 41494985 10.1093/nar/gkaf1333 PMC12865454

[21] Bravo-Hernandez M, Mazur C, Chen H et al. Transient acute neuronal activation response caused by high concentrations of oligonucleotides in the cerebral spinal fluid. Nucleic Acids Res. 2026;54:gkag057. 10.1093/nar/gkag057. 41633500 PMC12867516

[22] Brashear A, Sweadner KJ, Haq I et al. ATP1A3-Related Disorder. GeneReviews. University of Washington, Seattle 2008. 20301294

[23] Williamson JR . G-quartet structures in telomeric DNA. Annu Rev Biophys Biomol Struct. 1994;23:703–30. 10.1146/annurev.bb.23.060194.003415. 7919797

[24] Pollak AJ, Cauntay P, Machemer T et al. Mechanism driven early stage identification and avoidance of antisense oligonucleotides causing TRL9 mediated inflammatory responses in Bjab cells. bioRxiv, 10.1101/2021.12.12.472280, 14 December 2021, preprint: not peer reviewed..

[25] Partridge W, Burel SA, Ferng A et al. Correlations between preclinical BJAB assay ranking of antisense drugs and clinical trial adverse events. Clin Transl Sci. 2023;16:575–80. 10.1111/cts.13476. 36631935 PMC10087069

[26] O’Rourke JG, Bachmann G, Mazur C et al. Reversable acute sedation response of phosphorothioate antisense oligonucleotides following local delivery to the central nervous system. bioRxiv, 10.1101/2025.02.13.638136, 17 February 2025, preprint: not peer reviewed.. PMC12865454 41494985

[27] Kuroda T, Yoshioka K, Mon SSL et al. Unraveling and controlling late-onset neurotoxicity of antisense oligonucleotides through strategic chemical modifications. Mol Ther Nucleic Acids. 2025;36:102692. 10.1016/j.omtn.2025.102692. 41467114 PMC12744863

[28] Shen W, De Hoyos CL, Migawa MT et al. Chemical modification of PS-ASO therapeutics reduces cellular protein-binding and improves the therapeutic index. Nat Biotechnol. 2019;37:640–50. 10.1038/s41587-019-0106-2. 31036929

[29] McCauley ME, Bennett CF. Antisense drugs for rare and ultra-rare genetic neurological diseases. Neuron. 2023;111:2465–8. 10.1016/j.neuron.2023.05.027. 37354903

[30] Zhang W, Mittal S, Thomas R et al. A toxic gain-of-function variant in MAPK8IP3 provides insights into JIP3 cellular roles. JCI Insight. 2025;10:e187199. 10.1172/jci.insight.187199. 40111412 PMC12016931

[31] Ziegler A, Carroll J, Bain JM et al. Antisense oligonucleotide therapy in an individual with KIF1A-associated neurological disorder. Nat Med. 2024;30:2782–6. 10.1038/s41591-024-03197-y. 39122967 PMC12010239

[32] Prakash TP, Graham MJ, Yu J et al. Targeted delivery of antisense oligonucleotides to hepatocytes using triantennary *N*-acetyl galactosamine improves potency 10-fold in mice. Nucleic Acids Res. 2014;42:8796–807. 10.1093/nar/gku531. 24992960 PMC4117763

[33] Duan N, Kravitz RL, Schmid CH. Single-patient (n-of-1) trials: a pragmatic clinical decision methodology for patient-centered comparative effectiveness research. J Clin Epidemiol. 2013;66:S21–8. 10.1016/j.jclinepi.2013.04.006. 23849149 PMC3972259

[34] McColgan P, Thobhani A, Boak L et al. Tominersen in adults with manifest huntington’s disease. N Engl J Med. 2023;389:2203–5. 10.1056/NEJMc2300400. 38055260

[35] Liang XH, Sun H, Shen W et al. Antisense oligonucleotides targeting translation inhibitory elements in 5' UTRs can selectively increase protein levels. Nucleic Acids Res. 2017;45:9528–46. 10.1093/nar/gkx632. 28934489 PMC5766168

[36] Liang X-h, Shen W, Sun H et al. Translation efficiency of mRNAs is increased by antisense oligonucleotides targeting upstream open reading frames. Nat Biotechnol. 2016;34:875–80. 10.1038/nbt.3589. 27398791

[37] Jafar-Nejad P, Powers B, Soriano A et al. The atlas of RNase H antisense oligonucleotide distribution and activity in the CNS of rodents and non-human primates following central administration. Nucleic Acids Res. 2021;49:657–73. 10.1093/nar/gkaa1235. 33367834 PMC7826274

[38] Vickers TA, Sabripour M, Crooke ST. U1 adaptors result in reduction of multiple pre-mRNA species principally by sequestering U1snRNP. Nucleic Acids Res. 2011;39:e71. 10.1093/nar/gkr150. 21415007 PMC3105408

[39] Hodges D, Crooke ST. Inhibition of splicing of wild-type and mutated luciferase-adenovirus pre-mRNAs by antisense oligonucleotides. Mol Pharmacol. 1995;48:905–18. 10.1016/S0026-895X(25)10549-X. 7476922

[40] U.S. Food and Drug Administration . Considerations for the use of the Plausible Mechanism Framework to Develop Individualized Therapies that Target Specific Genetic Conditions with Known Biological Cause. https://www.fda.gov/regulatory-information/search-fda-guidance-documents/considerations-use-plausible-mechanism-framework-develop-individualized-therapies-target-specific. Date accessed 09 March, 2026.

[41] Crooke ST . Addressing the needs of nano-rare patients. Nucleic Acid Ther. 2025;35:51–4. 10.1089/nat.2024.0091 39876716

