# Supplementary material for: Addressing the needs of nano-rare patients: the n-Lorem experience
Source: Nucleic Acids Res. 2026 Jun 2;54(10):gkag504. doi: 10.1093/nar/gkag504 (PMC13227102; doi:10.1093/nar/gkag504)
Supplement: gkag504_Supplemental_Files [file gkag504_supplemental_files.zip › SuppFig1.pptx]

## Slide 1
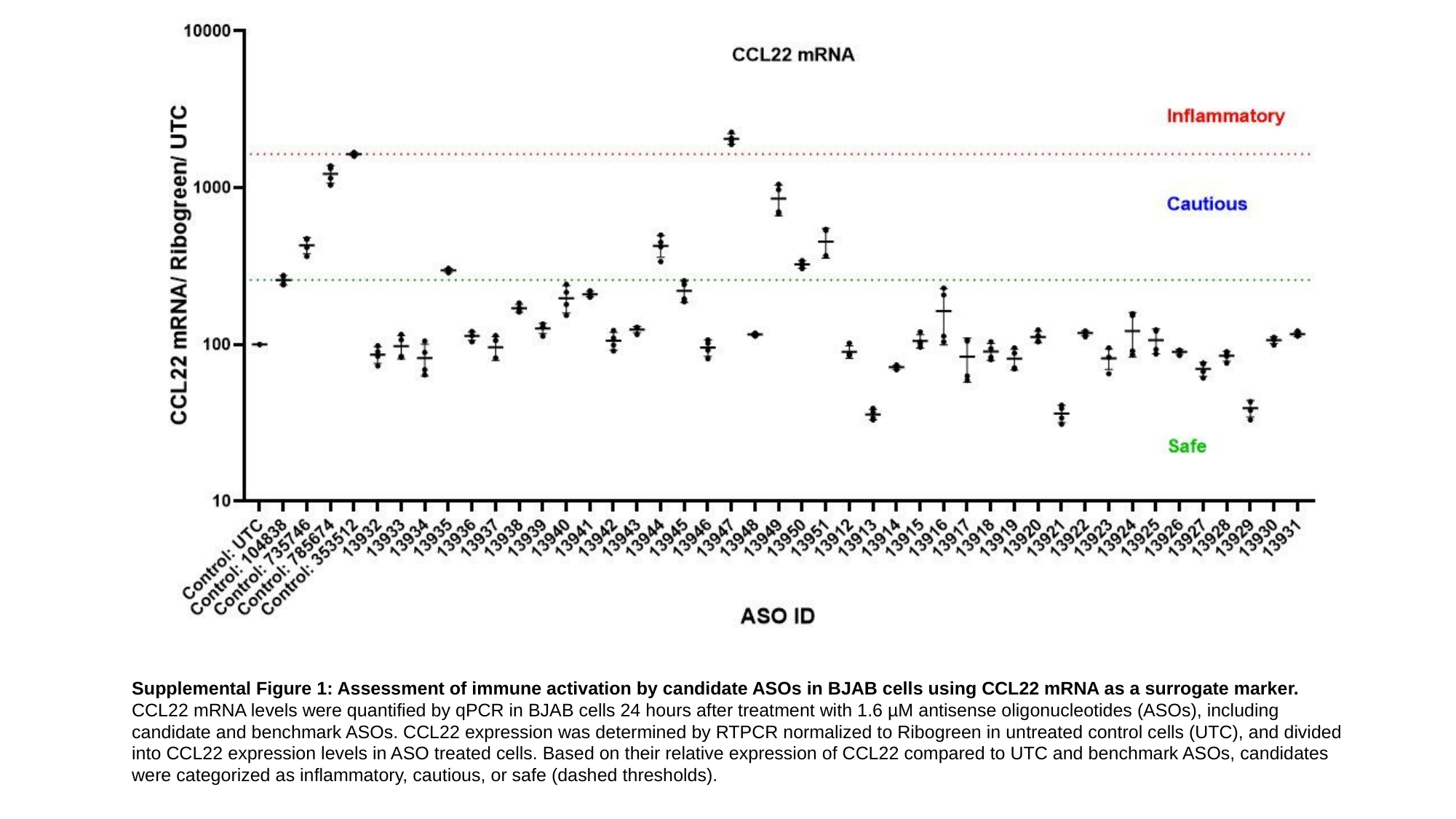

Supplemental Figure 1: Assessment of immune activation by candidate ASOs in BJAB cells using CCL22 mRNA as a surrogate marker.
CCL22 mRNA levels were quantified by qPCR in BJAB cells 24 hours after treatment with 1.6 µM antisense oligonucleotides (ASOs), including candidate and benchmark ASOs. CCL22 expression was determined by RTPCR normalized to Ribogreen in untreated control cells (UTC), and divided into CCL22 expression levels in ASO treated cells. Based on their relative expression of CCL22 compared to UTC and benchmark ASOs, candidates were categorized as inflammatory, cautious, or safe (dashed thresholds).
