# Supplementary material for: Addressing the needs of nano-rare patients: the n-Lorem experience
Source: Nucleic Acids Res. 2026 Jun 2;54(10):gkag504. doi: 10.1093/nar/gkag504 (PMC13227102; doi:10.1093/nar/gkag504)
Supplement: gkag504_Supplemental_Files [file gkag504_supplemental_files.zip › Supplemental Materials.docx]

**Supplemental Material -** Key Quality Assurance Committees

**Access to Treatment Committee (ATTC)**

Advise on whether a patient is appropriate to treat with an ASO.

| **n-Lorem Representatives** | **External Experts** |
| --- | --- |
| **Laurence Mignon, Ph.D.,** Executive Director, Clinical Development – *Co-chair*  **Julie Douville, Ph.D.,** Vice President, ASO Discovery and Development  **Sarah Glass, Ph.D.,** Chief Operating Officer  **Megan Knutsen, M.S.,** Senior Director, Clinical Operations and Project Management  **Konstantina Skourti-Stathaki, Ph.D.,** Executive Director, ASO Discovery and Research  **Amy Williford, Ph.D.,** Vice President, Foundation Development and External Relations | **Joseph G. Gleeson, M.D.,** Chief Medical Officer at n-Lorem and Rady Professor, Neurosciences and Pediatrics, University of California, San Diego *– Co-chair*  **C. Frank Bennett, Ph.D.,** Executive Vice President, Chief Scientific Officer, Ionis Pharmaceuticals Inc.  **Allison Bateman-House, M.D., Ph.D.,** Assistant Professor of Population Health at NYU Grossman School of Medicine  **Lauren Black, Ph.D.,** Distinguished Scientist at Charles River Laboratories  **Shyamanga Borooah, M.B.B.S., Ph.D.,** Assistant Clinical Professor of Ophthalmology at the Shiley Eye Institute at UC San Diego Health  **Jeff Carroll, Ph.D.,** Associate Professor in the Department of Neurology at the University of Washington  **James J. Dowling, M.D., Ph.D.,** Director, Penn Neurogenetics Therapy Center  **Toby Ferguson, M.D., Ph.D.,** Chief Medical Officer, Voyager Therapeutics  **Richard Finkel, M.D.,** Director, Experimental Neuroscience Program, and Endowed Chair in Neurotherapeutics at St. Jude Children’s Research Hospital  **Kenneth ‘Kurt’ Fischbeck, M.D.,** NIH Distinguished Investigator, Neurogenetics Branch  **Jeffrey Noebels, M.D., Ph.D.,** Cullen Chair in Neurogenetics and Professor of Neurology, Neuroscience, and Molecular and Human Genetics at Baylor College of Medicine  **Francis Sessions Cole, III, M.D.,** Professor of Pediatrics at Washington University School of Medicine in St. Louis  **Neil Shneider, M.D., Ph.D.,** Claire Tow Associate Professor of Motor Neuron Disorders and Director of the Eleanor and Lou Gehrig ALS Center at Columbia University |

**Research Management Committee (RMC)**

Advise on whether an ASO is satisfactory to advance to the clinic.

| **n-Lorem Representatives** | **External Experts** |
| --- | --- |
| **Stanley T. Crooke, M.D., Ph.D.,** Founder, Chief Executive Officer and Chairman of the Board - *Chair*  **Sarah Glass**, Ph.D., Chief Operating Officer  **Julie Douville**, Ph.D., Vice President, ASO Discovery and Development  **Laurence Mignon**, Ph.D., Executive Director, Clinical Development  **Megan Knutsen**, M.S., Senior Director, Clinical Operations and Project Management  **Konstantina Skourti-Stathaki**, Ph.D., Executive Director, ASO Discovery and Research  **Amy Williford**, Ph.D., Vice President, Foundation Development and External Relations | **Joseph G. Gleeson, M.D.,** Chief Medical Officer at n-Lorem and Rady Professor, Neurosciences and Pediatrics, University of California, San Diego  **C. Frank Bennett, Ph.D.,** Executive Vice President, Chief Scientific Officer, Ionis Pharmaceuticals Inc.  **Richard Geary, Ph.D.,** Strategic Advisor Consultant, MedDev Insights, LLC., former Executive Vice President and Chief Development Officer at Ionis Pharmaceuticals, Inc.  **Scott Henry, Ph.D.,** Senior Vice President, Preclinical Development, Ionis Pharmaceuticals |

**Study Treatment and Assessment Review Committee (STAR)**

Advise about appropriateness of treatment goals, prespecified clinical endpoints and the clinical protocol.

| **n-Lorem Representatives** | **External Experts** |
| --- | --- |
| **Sarah Glass**, Ph.D., Chief Operating Officer  **Laurence Mignon**, Ph.D., Executive Director, Clinical Development | **Joseph G. Gleeson, M.D.,** Chief Medical Officer at n-Lorem and Rady Professor, Neurosciences and Pediatrics, University of California, San Diego - *Chair*  **Elizabeth M. Berry Kravis, M.D., Ph.D.,** Professor of Pediatrics, Neurological Sciences, and Biochemistry at Rush University Medical Center - *Co-Chair*  **Wendy Chung, M.D., Ph.D.,** Chair of Pediatrics at Boston Children's Hospital and Harvard Medical School  **Richard Finkel, M.D.,** Director, Experimental Neuroscience Program, and Endowed Chair in Neurotherapeutics at St. Jude Children’s Research Center  **Eugene Schneider, M.D.,** Executive Vice President, Chief Clinical Development and Operations Officer at Ionis Pharmaceuticals |

**Data Safety Monitoring Board (DSMB)**

Monitors safety of all AOSs in the clinic and aggregate safety. Also, confirms assessment of benefit.

| **n-Lorem Representatives** | **External Experts** |
| --- | --- |
| **Sarah Glass**, Ph.D., Chief Operating Officer  **Laurence Mignon**, Ph.D., Executive Director, Clinical Development  **He Pu, Ph.D.,** Senior Clinical Data Scientist | **Eugene Schneider, M.D.,** Executive Vice President, Chief Clinical Development and Operations Officer at Ionis Pharmaceuticals *- Chair*  **Joseph G. Gleeson, M.D.,** Chief Medical Officer at n-Lorem and Rady Professor, Neurosciences and Pediatrics, University of California, San Diego  **Elizabeth M. Berry Kravis, M.D., Ph.D.,** Professor of Pediatrics, Neurological Sciences, and Biochemistry at Rush University Medical Center  **Wendy Chung, M.D., Ph.D.,** Chair of Pediatrics at Boston Children's Hospital and Harvard Medical School  **Richard Finkel, M.D.,** Director, Experimental Neuroscience Program, and Endowed Chair in Neurotherapeutics at St. Jude Children’s Research Center  **Lisa R. Grillone, Ph.D.,** PharmaQuest Associates  **Francis Sessions Cole, III, M.D.,** Professor of Pediatrics at Washington University School of Medicine in St. Louis |
